# Narrowing sex differences in pharmacotherapy following invasive angiography for coronary artery disease between 2017 and 2024

**DOI:** 10.1093/ehjopen/oeag099

**Published:** 2026-06-08

**Authors:** Andrew J Sullivan, Zin Tun, Margaret Siu, Manisha Rai, Gloria Antoun, Nandini Rawat, Rebecca Walker, Alexandros Kythreotis, Abdelmajid Al Jariri, Asad Shabbir, Shane Cashin, Anthony Mathur, Daniel A Jones, Ajay Gupta, Krishnaraj S Rathod, Amrita Ahluwalia

**Affiliations:** Barts and the London Faculty of Medicine and Dentistry, Queen Mary University of London, Charterhouse Square, London, EC1M 6BQ, UK; Barts Heart Centre, Barts Health NHS Trust, London, West Smithfield, London, EC1A 7BE, UK; Barts Heart Centre, Barts Health NHS Trust, London, West Smithfield, London, EC1A 7BE, UK; Barts and the London Faculty of Medicine and Dentistry, Queen Mary University of London, Charterhouse Square, London, EC1M 6BQ, UK; Barts and the London Faculty of Medicine and Dentistry, Queen Mary University of London, Charterhouse Square, London, EC1M 6BQ, UK; Barts and the London Faculty of Medicine and Dentistry, Queen Mary University of London, Charterhouse Square, London, EC1M 6BQ, UK; Barts and the London Faculty of Medicine and Dentistry, Queen Mary University of London, Charterhouse Square, London, EC1M 6BQ, UK; Barts and the London Faculty of Medicine and Dentistry, Queen Mary University of London, Charterhouse Square, London, EC1M 6BQ, UK; Barts and the London Faculty of Medicine and Dentistry, Queen Mary University of London, Charterhouse Square, London, EC1M 6BQ, UK; Barts Heart Centre, Barts Health NHS Trust, London, West Smithfield, London, EC1A 7BE, UK; Barts and the London Faculty of Medicine and Dentistry, Queen Mary University of London, Charterhouse Square, London, EC1M 6BQ, UK; Barts Heart Centre, Barts Health NHS Trust, London, West Smithfield, London, EC1A 7BE, UK; Barts and the London Faculty of Medicine and Dentistry, Queen Mary University of London, Charterhouse Square, London, EC1M 6BQ, UK; Barts Heart Centre, Barts Health NHS Trust, London, West Smithfield, London, EC1A 7BE, UK; Barts and the London Faculty of Medicine and Dentistry, Queen Mary University of London, Charterhouse Square, London, EC1M 6BQ, UK; Barts Heart Centre, Barts Health NHS Trust, London, West Smithfield, London, EC1A 7BE, UK; Barts and the London Faculty of Medicine and Dentistry, Queen Mary University of London, Charterhouse Square, London, EC1M 6BQ, UK; Barts and the London Faculty of Medicine and Dentistry, Queen Mary University of London, Charterhouse Square, London, EC1M 6BQ, UK; Barts Heart Centre, Barts Health NHS Trust, London, West Smithfield, London, EC1A 7BE, UK; Barts and the London Faculty of Medicine and Dentistry, Queen Mary University of London, Charterhouse Square, London, EC1M 6BQ, UK

**Keywords:** Coronary artery disease, Pharmacotherapy, Sex characteristics, Myocardial infarction

## Abstract

**Introduction:**

Women who experience myocardial infarction (MI) and undergo invasive angiography, experience higher morbidity and mortality compared to age-matched male counterparts. The prognostic benefit of optimal medical therapy (OMT) following MI is well established; however, treatment bias has been evidenced historically between the sexes. We explored sex differences in prescribing trends of OMT following invasive angiography for obstructive CAD at a high throughput regional cardiac centre.

**Methods:**

We determined discharge medication received by females and males undergoing invasive angiography in 2017, 2019, 2022, and 2024 with obstructive CAD (angiographic lesion ≥50% luminal diameter). Logistic regression was used to determine differences in the main cohort and in subgroups by clinical diagnosis (ACS, STEMI, NSTEMI, stable angina) and age (<55 or ≥55 years). This latter age cut-off to explore pre- and post-menopause trends, respectively.

**Results:**

10 591 patient attendances (22.3% female, *n* = 2360) were included in the analysis. In the overall cohort, women were less likely to receive β-blockers (*P* = 0.002), ACE-I/ARB (*P* = 0.002), and high potency P2Y_12_ inhibitors (*P* < 0.001) compared to males. In ACS, similar patterns were observed for β-blockers and high potency P2Y_12_ inhibitors, women ≥55 years were less likely to receive high intensity statin (HIS). However, we show significant improvements in the prescribing of β-blockers (*P* = 0.018) in women over time, and a trend towards improved prescribing of high potency P2Y_12_ inhibitors (*P* = 0.085) in ACS.

**Conclusion:**

These findings demonstrate welcome improvements in equitable prescribing practices for OMT post angiography and highlight the importance of reviewing prescribing practices to ensure evidencing of success in implementing best practice guidelines.

## Graphical Abstract

**Graphical Abstract:**
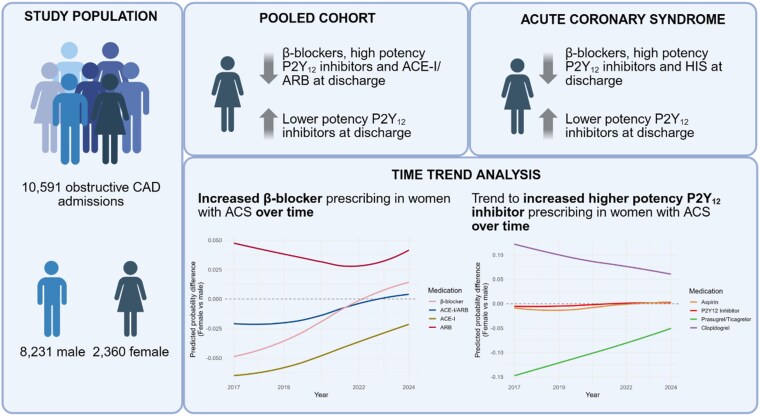

## Introduction

Coronary artery disease (CAD) remains the main cause of death in women worldwide, despite advances in treatment.^1^ In addition, data suggests that women have worse outcomes following percutaneous coronary intervention (PCI) and stent placement in acute coronary syndromes (ACS) compared to their age-matched male counterparts.^2,3^ These poorer outcomes seem to be particularly prevalent in post-menopausal women with STEMI.^4^ Globally, studies examining these issues have highlighted that a major challenge in understanding the causes of this disparity is the persistent under-representation of women in contemporary cardiovascular clinical trials, even after accounting for the lower prevalence of CAD in women compared with men.^5^ Thus, indicating that the evidence base is more directed towards men. In addition, evidence has demonstrated disparities in the application of treatment pathways in CAD. Studies have shown that women experience delays in diagnosis, undergo less revascularization and experience delays in revascularization.^6,7^ Similar disparities have also been evidenced in aftercare, with women less likely to receive risk modifying medical therapies and cardiac rehabilitation following PCI.^8–10^

Optimal medical therapy (OMT), routinely recommended for patients with CAD to reduce future risk, in particular after ACS,^11^ involves the combination of aspirin, P2Y_12_ inhibitors, statin, β-blocker, and angiotensin converting enzyme inhibitor (ACE-I)/angiotensin receptor blocker (ARB). Published datasets have suggested that a key driver of the poorer outcomes for women post-PCI (in STEMI) may relate to under-prescription of OMT in women.^9,10^ However, it remains unknown whether under-prescription of OMT is prevalent for all sub-groups of CAD, i.e. STEMI, NSTEMI, stable angina. This issue is an important one to address since evidence indicates that outcomes for women treated for NSTEMI and stable angina are worse too.^12,13^

Sex disparities in cardiovascular care are now widely recognized, and there is growing consensus that ongoing evaluation with up-to-date analyses of temporal trends is essential to determine whether meaningful progress is being made in narrowing the gap.^1^ There is therefore a need for a comprehensive analysis that brings these points together in a single patient cohort.^12,13^ Thus, in this study we determined whether the raised awareness of sex difference in OMT prescriptions has coincided with improvements in the prescribing of OMT drugs at discharge following invasive angiography in patients with CAD. This has been assessed over a period spanning 2017–2024 in patients attending a large tertiary Cardiac Centre operating within the UK NHS healthcare system. In addition, we have explored whether prescribing practices differed between pre- and post-menopausal women, according to the underlying diagnosis of CAD (i.e. STEMI, NSTEMI, and stable angina), by ethnicity and by socio-economic status. To our knowledge, our assessment provides a unique comprehensive analysis of the issues in CAD over a time period (2017–2024) when major initiatives to raise awareness of sex differences in CAD were initiated.^1,14,15^

## Methods

### Study design

This is a single centre study of patient attendances to the large tertiary Barts Heart Centre, Barts Health NHS Trust, London, UK, for invasive angiography. This study utilized data from the Barts Revascularisation Registry (NCT05255705, EDGE ID: 142567) from the years 2017, 2019, 2022, and 2024. The study was conducted in compliance with the Declaration of Helsinki. Medications prescribed on discharge from hospital for patients undergoing invasive angiography were compared. Patients were identified using the approved Barts Revascularisation Registry (angiography database) in which all patients who undergo invasive angiography at Barts Heart Centre are prospectively logged. Variables recorded include demographics (age, sex, ethnicity), medical history (hypertension, hypercholesterolaemia, diabetes, smoking status, previous MI, previous PCI, previous CABG), left ventricular ejection fraction, and procedural information (vessel[s] and degree of lesion, interventions performed). Additional information including clinical measurements such as blood pressure and heart rate were obtained from the electronic patient health record. Information on individual medical OMT drugs and their dose prescribed (aspirin, P2Y_12_ inhibitors, statins, β-blockers, ACE-I, and ARBs) were extracted from the medication information listed in discharge summaries in the electronic patient record. This data was inputted into a dedicated database.

**Figure 1:**
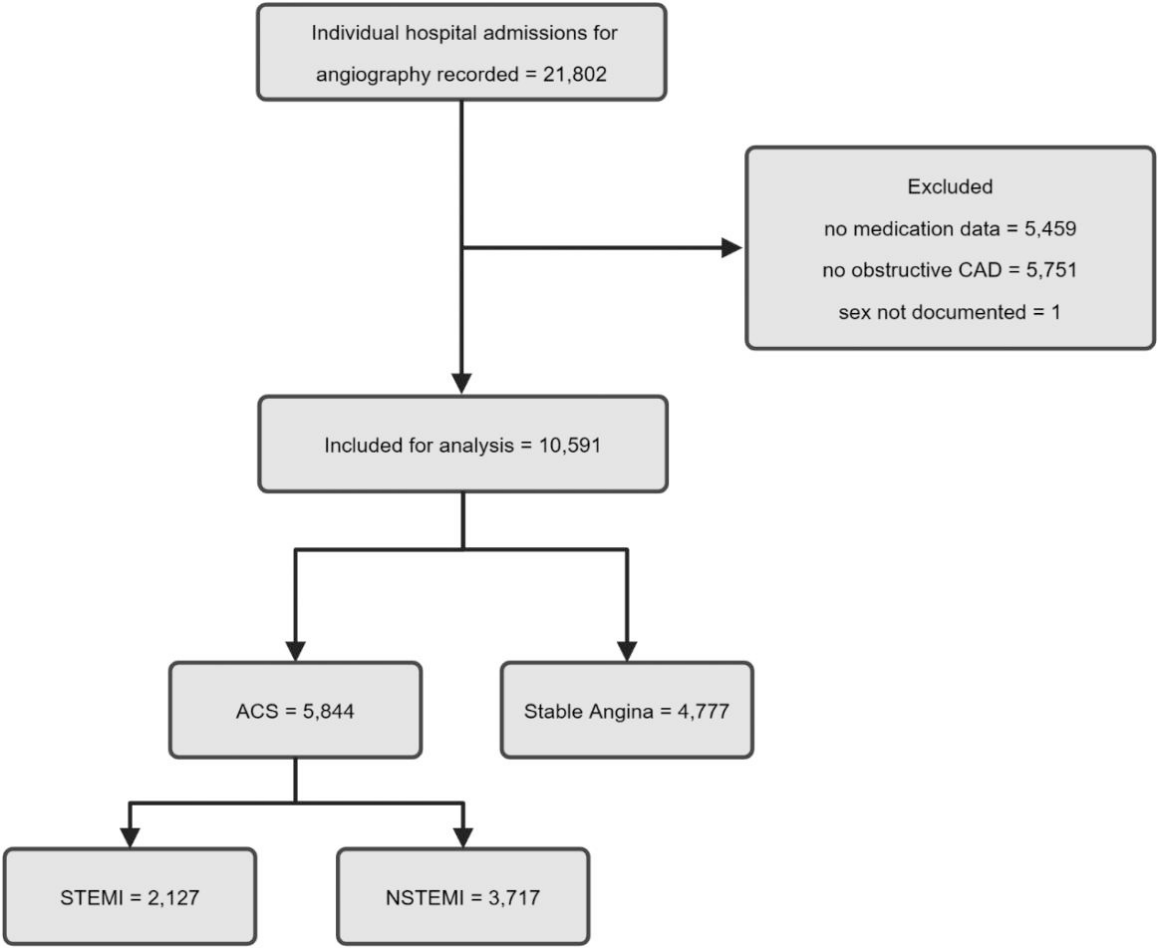
Strobe diagram.

For analysis we included only patients with obstructive CAD (defined as an epicardial lesion ≥50%).^11^ Records where no medication data were available were excluded, as were records where the sex was missing. *Figure 1* shows a STROBE diagram for this cohort. Data was collected from the years chosen to determine prescribing trends over time, balancing this with the constraints of manual data collection. We opted not to include 2020–2021 due to the impact of the COVID pandemic where regular care was significantly disrupted and prescribing patterns would not reflect usual trends.^16^

### Statistical analysis

Statistical analysis was conducted using R Studio 2023.12.0 + 369. Categorical data were compared using the χ^2^ or Fisher’s exact test. Students *t*-test and Mann–Whitney U test were used to compare normally and non-normally distributed continuous data respectively. The Shapiro–Wilk test was used to confirm the normality of continuous data where relevant.

For the purposes of analysis, medication data was categorized according to the main drug classes. ACE-I and ARBs were analysed separately and as combined ACE-I/ARB. P2Y_12_ inhibitors were combined into one category for analysis but also separated out into higher potency (ticagrelor and prasugrel) and lower potency (clopidogrel) agents. Statin therapy was also stratified into ‘any’ statin and high intensity statin (HIS) treatment, as HIS is recommended for optimal risk reduction and there exists evidence suggesting that women are less likely to receive HIS, or up-titration of statin dose as part of OMT according to guidelines.^11,17,18^ The definition of HIS used was statin therapy that will result in ≥50% reduction in LDL-C, which corresponds to atorvastatin 40–80 mg daily or rosuvastatin 20–40 mg daily.^19^

### Multivariate logistic regression

To determine sex differences in pharmacotherapy multivariable binomial logistic regression modelling was used. The effect of biological sex was assessed on whether patients received each class and OMT drugs, adjusting for confounders (age, BMI, diabetes, hypertension, hypercholesterolaemia, previous MI, previous PCI, previous CABG, and current PCI). An interaction term between sex and age was included in the model to account for potential effect modification. Age was centred for the purposes of analysis. Where continuous data for covariates was missing this was imputed using multiple imputations by chained equations to avoid data loss for the model. Where categorical data was missing or unknown, this was assigned as an unknown category which was included in the model. Results from the modelling are reported as odds ratios (ORs) with 95% confidence intervals (Cis), a *P* value of <0.05 was taken to indicate significant differences.

All patient attendances were included in a pooled cohort for analysis to describe baseline characteristics and explore general associations. However, given the heterogeneity of the pooled population and the clinical relevance of OMT in ACS, sub-group analysis was additionally conducted according to key clinical categories of ACS, STEMI, NSTEMI, and stable angina. Exploratory subgroup analysis according to age group (<55 years or ≥55 years) was also performed as a surrogate for confirmed menopause. An age cut-off of 55 years was used, since the average age of menopause is 51 years, and menopause is generally complete by 55 years. Therefore, 55 years was used to estimate patterns of prescribing in pre- and post-menopause groups. Other registry-based studies have similarly utilized this age cut-off.^4,20^

### Socio-demographic analysis

Further exploratory subgroup analysis to assess for sex differences in different socio-demographic groups, by ethnicity and socio-economic status was conducted. Ethnicity data was obtained from the electronic patient health record and grouped into categories: Black, Caucasian, South Asian (Indian, Pakistani, Bangladeshi), other Asian, mixed ethnicity, and not stated. The not stated category included a combination of patients listed on the NHS record as ‘other ethnicity’, ‘ethnicity unknown’, and ‘ethnicity not stated’. Similarly, sex differences according to socio-economic status was performed using the Index of Multiple Deprivation (IMD) 2019 score. The IMD score is an official measure of relative deprivation for small localities in England. Patients’ postcodes were used to match to IMD rankings and rankings were grouped into quintiles for analysis, with 1 being areas of highest deprivation and 5 being areas of lowest deprivation. IMD scores have been used widely to search for relationships between socio-economic factors and health.^21^

### Sensitivity analysis

An exploratory sensitivity analysis was carried out to determine the robustness of findings in the context of other relevant confounders. Heart rate (<60/≥60), systolic blood pressure (<120/≥120), left ventricular ejection fraction (LVEF) (preserved/reduced), haemoglobin level (<120/≥120), and estimated glomerular filtration rate (eGFR) (<60/≥60) were included individually as interaction terms in our main logistic regression model to determine sex-specific effects. Statistics are reported as the odds ratio and 95% confidence interval of the interaction term between sex and secondary covariate with *P* < 0.05 considered significant. Due to the possibility of within-patient clustering arising from repeat attendances, a further sensitivity analysis was performed in which results from the main attendance-level models were compared with models restricted to index presentations only. Odds ratios, directions of effect, and *P* values were compared to assess whether the findings changed materially.

### Time trend analysis

To determine differences over time, joinpoint logistic regression was performed using the same covariates from the main model with the addition of time from start determined by the date of angiogram procedure. A *P* value of <0.05 for the interaction term between sex and time from start was taken to signify a significant change over time. The difference between the predicted probabilities between the sexes of each medication type was plotted to visualize changes over time.

## Results

### Demographics

In the final analysis, 10 591 patient attendances (9446 unique patients) were included, the demographics of which are outlined in *Table 1*. Females were on average older than males (*P* < 0.001). There was a higher proportion of female patients with black ethnicity (*P* < 0.001) and white ethnicity (*P* = 0.003) compared to the proportions in the male group, whilst a higher proportion of male patients are of South Asian ethnicity (*P* < 0.001). Proportionately more females had hypertension and diabetes (*P* < 0.001), however females were less likely to have a previous cardiovascular history (*P* < 0.001). Females were more likely to have a normal left ventricular ejection fraction (*P* = 0.002), and there was no difference in the proportions between the sexes having current coronary intervention. Supplementary material online, *Tables S1–S4* show a breakdown of the demographics according to clinical diagnosis (ACS, STEMI, NSTEMI, and stable angina).

**Table 1:** Demographics of the study cohort by sex

	All	Female	Male	*P* value
Patients	10 591	2360 (22.3%)	8231 (77.7%)	
Age (years)	62.5 (±12.04)	66.0 (±11.8)	61.4 (±11.9)	<0.001
**Ethnicity**				
Black	531 (5.0%)	180 (7.6%)	351 (4.3%)	<0.001
Caucasian	4787 (45.2%)	1140 (48.3%)	3647 (44.3%)	0.001
South Asian	2963 (28.0%)	593 (25.1%)	2370 (28.8%)	0.001
Other Asian	912 (8.6%)	156 (6.6%)	756 (9.2%)	<0.001
Mixed	126 (1.2%)	34 (1.4%)	92 (1.1%)	0.243
Not stated	1272 (12.0%)	257 (10.9%)	1015 (12.3%)	0.062
**Co-morbidities**				
Hypertension	6250 (59.0%)	1535 (65.0%)	4715 (57.3%)	<0.001
Hypercholesterolaemia	6141 (58.0%)	1381 (58.5%)	4760 (57.8%)	0.806
Diabetes	3934 (37.5%)	1033 (43.9%)	2901 (35.6%)	<0.001
Previous MI	3071 (29.0%)	539 (22.8%)	2532 (30.8%)	<0.001
Previous PCI	3247 (30.7%)	572 (24.2%)	2675 (32.5%)	<0.001
Previous CABG	894 (8.4%)	145 (6.1%)	749 (9.1%)	<0.001
**Intervention**				
PCI with stent	7042 (66.5)	1547 (65.6)	5495 (66.8)	0.284
PCI with no stent	885 (8.4)	189 (8.0)	696 (8.5)	0.516
**LV ejection fraction**				
≥50%	5024 (47.4)	1161 (49.2)	3863 (46.9)	0.055
30–49%	1714 (16.2)	335 (14.2)	1379 (16.8)	0.003
<30%	494 (4.7)	88 (3.7)	406 (4.9)	0.017
Not stated	3359 (31.7)	776 (32.9)	2583 (31.4)	0.175
**Clinical measurements**				
BMI (Kg/m^2^)	28.0 (± 6.0)	28.2 (± 6.2)	27.9 (± 6.0)	0.116
Heart rate (b.p.m.)	70.0 (±11.3)	70.85 (±11.0)	69.8 (±11.4)	0.011
Systolic blood pressure (mmHg)	121.9 (±18.9)	122.1 (±19.8)	121.78 (±18.7)	0.616
Diastolic blood pressure (mmHg)	70.0 (±10.0)	67.6 (±9.7)	70.72 (±9.9)	<0.001
Haemoglobin (g/L)	135.67 (±18.60)	123.94 (±16.58)	138.95 (±17.79)	<0.001
eGFR (mL/min/1.73m^2^)				
≥60	2997 (28.3%)	597 (25.3%)	2400 (29.2%)	<0.001
<60	824 (7.8%)	240 (10.2%)	584 (7.1%)	<0.001
Not stated	6770 (63.9%)	1523 (64.5%)	5247 (63.7%)	0.498

Statistics are expressed as number and % in brackets of the group for categorical data. For continuous data mean ± SD is reported. Categorical data were compared using the χ^2^ test. Normally distributed continuous data were compared using a *t*-test and non-normally distributed continuous data compared using the using the Mann–Whitney U test.

### Unadjusted data

Unadjusted data showing the overall number of medication prescriptions by group is displayed in *Table 2*. This shows that a lower proportion of females received all main OMT drug groups compared to males, except for the ‘any statin’ category (*P* = 0.063). Despite this, less women received HIS (*P* < 0.001). A lower proportion of women were prescribed prasugrel and ticagrelor as a P2Y_12_ inhibitor (*P* < 0.001) with a greater proportion of women receiving clopidogrel compared to men (*P* < 0.001). Fewer women received ACE-I (*P* < 0.001) but more women received ARB (*P* < 0.001). A further breakdown of unadjusted data by diagnosis is available in the supplement (see Supplementary material online, *Tables S5–S8*).

**Table 2:** Unadjusted medication prescription at discharge following invasive angiography by sex in all patients

Medication	All	Female	Male	*P* value
**Aspirin**	9767 (92.2%)	2149 (91.1%)	7618 (92.6%)	0.019
**P2Y12 inhibitor**	8980 (84.8%)	1957 (82.9%)	7023 (85.3%)	0.005
Clopidogrel	5423 (51.2%)	1356 (57.5%)	4067 (49.4%)	<0.001
Prasugrel & Ticagrelor	3821 (36.1%)	651 (27.6%)	3170 (38.5%)	<0.001
**Any statin**	9962 (94.1%)	2201 (93.3%)	7761 (94.3%)	0.063
High intensity statin	9163 (86.5)	1975 (83.7)	7188 (87.3)	<0.001
**β-blocker**	8848 (83.6%)	1907 (80.8%)	6941 (84.3%)	<0.001
**ACE-I or ARB**	8160 (77.1%)	1751 (74.2%)	6409 (77.9%)	<0.001
ACE-I	6603 (62.4%)	1312 (55.6%)	5291 (64.3%)	<0.001
ARB	1639 (15.5%)	464 (19.7%)	1175 (14.3%)	<0.001

Statistics are expressed as the absolute number and % in brackets of the group. χ^2^ test was used to determine differences between groups.

### Pooled multivariate analysis

**Figure 2:**
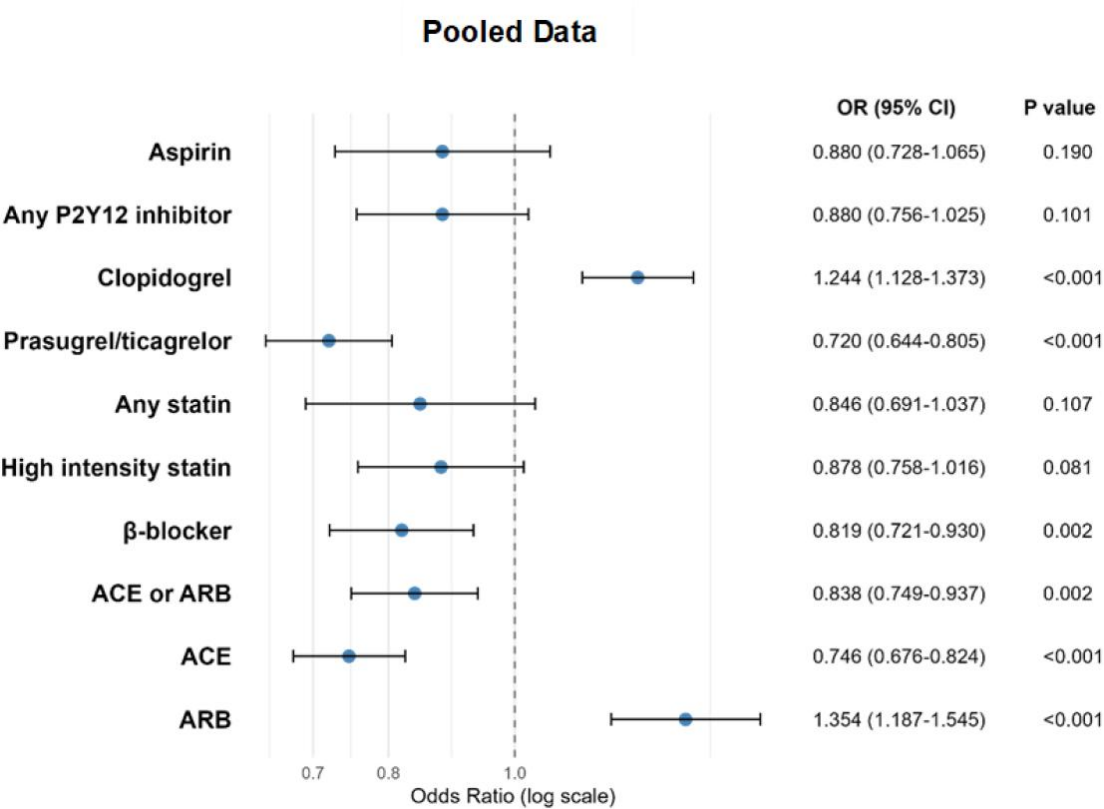
Multivariate logistic regression analysis* for sex differences in OMT prescribing across all patients (*n* = 10591, female = 2360). Statistics are displayed as odds ratios and 95% confidence intervals. Reference population = males, therefore odds ratio <1 taken as reduced in females. *Adjusted for age, BMI, diabetes, hypertension, hypercholesterolaemia, previous MI, previous PCI, previous CABG, and current PCI.

Pooled multivariate analysis of all patients demonstrated several differences in prescribing of OMT between males and females (*Figure 2*). Females were less likely to receive β-blockers (OR = 0.82 [0.72–0.93], *P* = 0.002) and ACE-I/ARBs (OR = 0.84 [0.75–0.94], *P* = 0.002). A pattern of reduced odds of ACE-I (OR = 0.75 [0.68–0.82], *P* < 0.001) and increased odds of ARB (OR = 1.35 [1.19–1.55], *P* < 0.001) was also noted in females. There is a non-significant trend towards reduced odds of P2Y_12_ inhibitor (OR = 0.88 [0.76–1.025], *P* = 0.101) and HIS (OR = 0.88 [0.76–1.016], *P* = 0.081) prescriptions in females and no significant differences in aspirin and statin prescriptions.

### Sub-group analysis by clinical diagnosis

**Figure 3:**
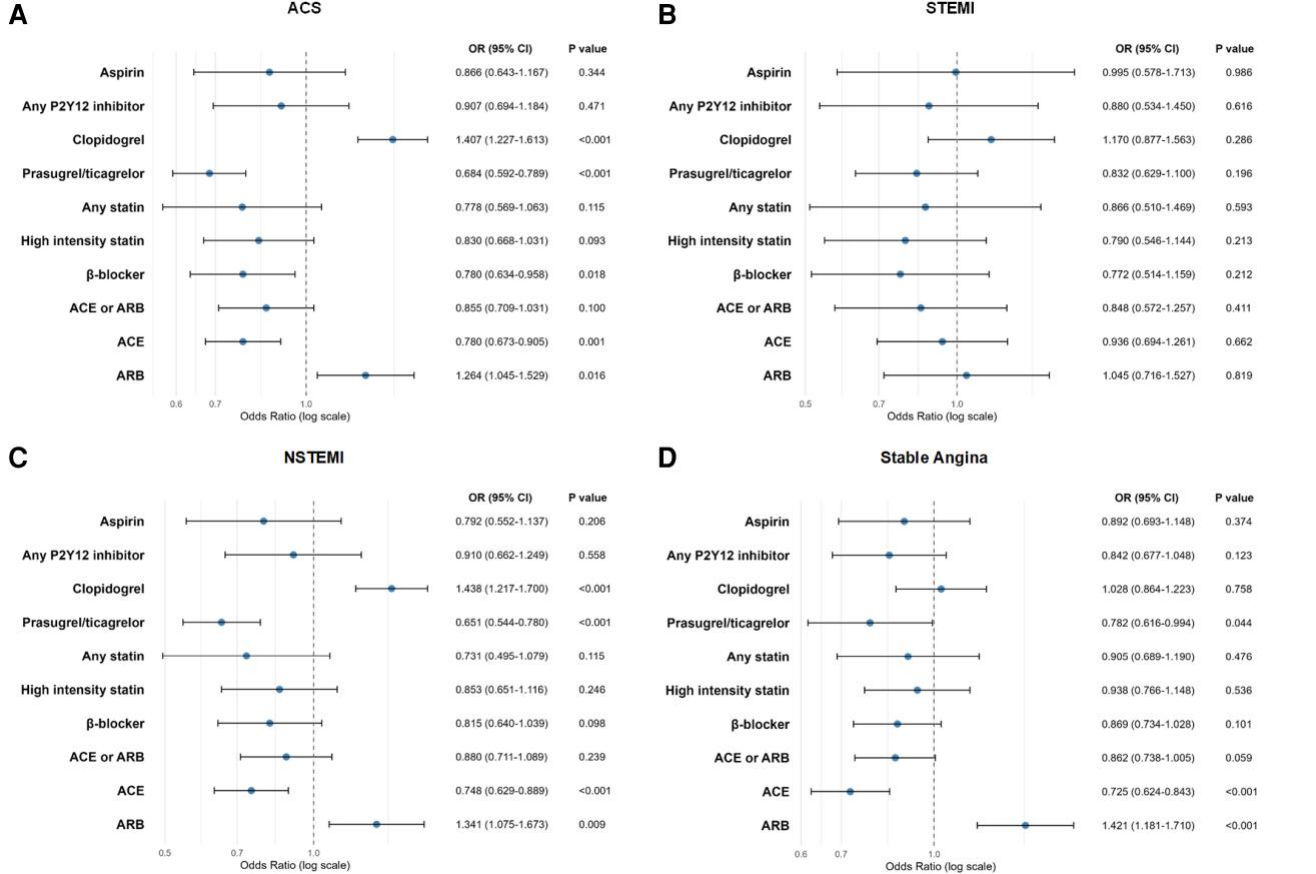
Multivariate logistic regression analysis* for sex differences in optimal medical therapy according to diagnosis. (*A*) ACS (*n* = 5844, female = 1314). (*B*) STEMI (*n* = 2127, female = 432). (*C*) NSTEMI (*n* = 3717, female = 882). (*D*) Stable angina (*n* = 4777, female = 1046). Statistics are displayed as odds ratios and 95% confidence intervals. Reference population = males, therefore odds ratio <1 taken as reduced in females. *Adjusted for age, BMI, diabetes, hypertension, hypercholesterolaemia, previous MI, previous PCI, previous CABG, and current PCI.

In patients with ACS (*Figure 3A*) females had a reduced odds of receiving β-blockers compared to males (OR = 0.78 [0.63–0.96], *P* = 0.019) and there was a significant interaction between sex and age relating to β-blocker prescriptions (OR = 1.02 [1.005–1.037], *P* = 0.010]. In ACS, we saw no sex difference in combined ACE-I/ARB prescriptions (OR = 0.86 [0.71–1.03], *P* = 0.100). However, when separated reduced ACE-I (OR = 0.78 [0.67–0.91], *P* = 0.001) prescriptions and increased ARB (OR [1.26 [1.05–1.53], *P* = 0.016) prescriptions in women became evident. Sub-group analysis according to diagnosis in ACS into STEMI (*Figure 3B*) and NSTEMI (*Figure 3C*) revealed that differences between the sexes were driven by the NSTEMI patients with no statistically significant differences in STEMI patients. Within the stable angina group (*Figure 3D*), females were less likely to receive ACE-I but more likely to receive an ARB, but as a combined group there was no statistical difference between the sexes (*Figure 3D*).

### Sub-group analysis by age

**Figure 4:**
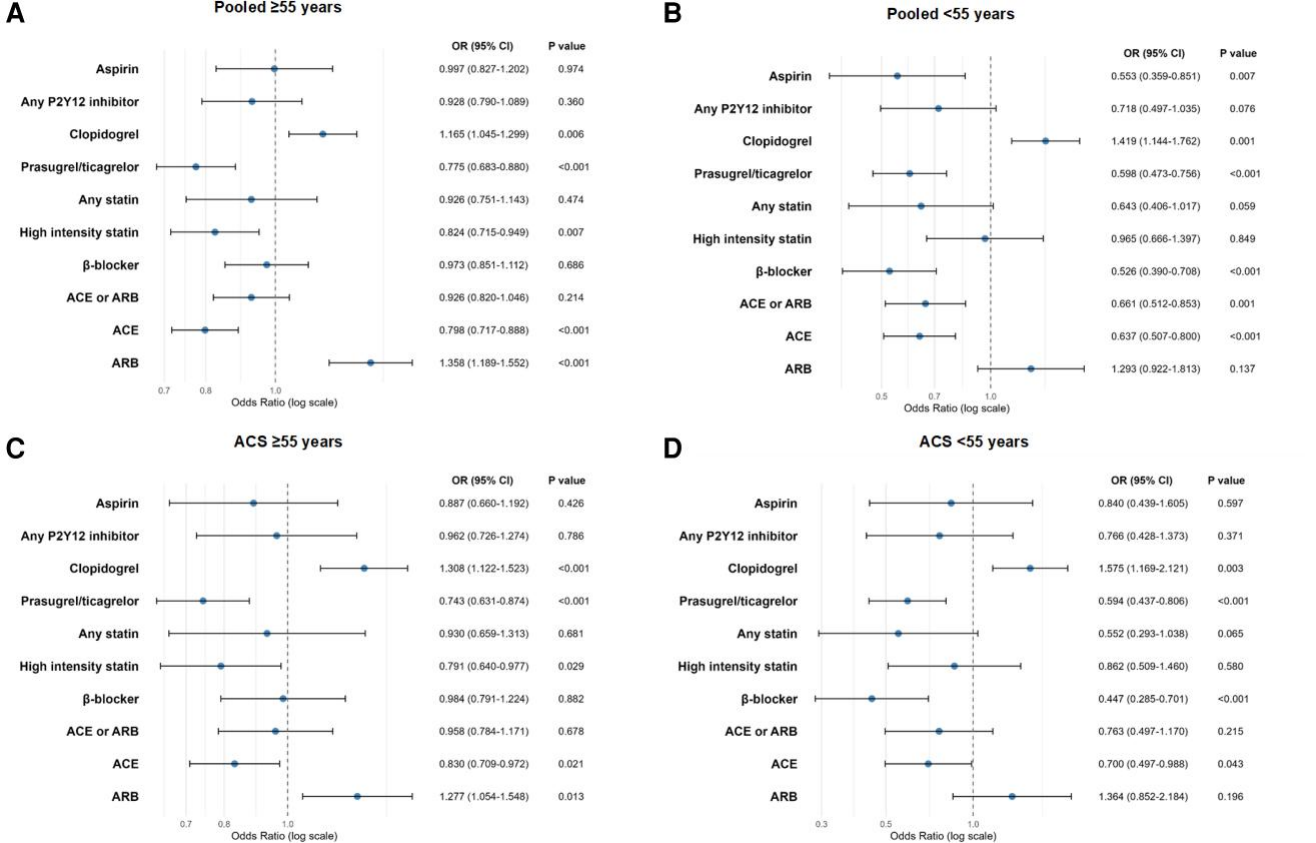
Multivariate logistic regression analysis* for sex differences in OMT according to age. (*A*) All patients ≥55 years (*n* = 7781, female = 1945). (*B*) All patients <55 years (*n* = 2810, female = 415). (*C*) ACS patients ≥55 years (*n* = 4084, female = 1058). (*D*) ACS patients <55 years (*n* = 1760, female = 256). Statistics are displayed as odds ratios and 95% confidence intervals. Reference population = males, therefore odds ratio <1 taken as reduced in females. *Adjusted for age, BMI, diabetes, hypertension, hypercholesterolaemia, previous MI, previous PCI, previous CABG, and current PCI.

By separation of the data into age bands corresponding to pre and post-menopausal age brackets, in the ≥55 years age group women received reduced HIS prescriptions compared to the men in the overall pooled cohort (*Figure 4A*) (OR = 0.82 [0.72–0.95], *P* = 0.007); and in the ACS sub-group (*Figure 4C*) (OR = 0.79 [0.64 −0.98], *P* = 0.029). In contrast, this effect was not observed in younger women. However, in the younger age group the proportion of women prescribed aspirin, β-blockers, ACEI, and ACEI/ARB combined, compared to men was significantly less. In the <55 years group, only the reduced proportions receiving β-blockers and ACEI remains in the ACS cohort of women (*Figure 4C*) (OR = 0.79 [0.64−0.98], *P* = 0.029. In the stable angina sub-group, women ≥55 years (see Supplementary material online, *Figure S1A*) were less likely to receive an ACEI (OR = 0.75 [0.65–0.88], *P* < 0.001) but more likely to receive an ARB (OR = 1.44 [1.20–1.74], *P* < 0.001), as a combined group however there was no difference in ACEI/ARB. Women <55 years with stable angina (see Supplementary material online, *Figure S1B*) were less likely to receive aspirin (OR = 0.39 [0.21–0.72], *P* = 0.002), β-blockers (OR = 0.66 [0.43–1.00], *P* = 0.047), and ACE (OR = 0.66 [0.46–0.96], *P* = 0.029].

### P2Y_12_ inhibitors

Interestingly, despite no significant difference in the proportion of patients receiving P2Y_12_ inhibitors in the overall cohort (*Figure 2*) or within sub-group of diagnosis (*Figure 3*), there were significant differences in type of inhibitor received between the sexes. In this cohort, females were less likely to receive a higher potency P2Y_12_ inhibitor (prasugrel/ticagrelor) (OR = 0.72 [0.64–0.81], *P* < 0.001) compared to males, with a corresponding increase in the odds of receiving a lower potency agent (clopidogrel) (OR = 1.24 [1.13–1.37], *P* < 0.001) (*Figure 2*). In the ACS sub-group, the same pattern was noted with reduced higher potency (OR = 0.68 [0.59–0.79], *P* < 0.001) and increased lower potency agents (clopidogrel) (OR = 1.41 [1.23–1.61], *P* < 0.001) (*Figure 3A*) prescribed. This sex difference in prescription of higher potency P2Y_12_ inhibitors was driven predominantly by differences in the NSTEMI patients (*Figure 3C*) (OR = 0.65 [0.54–0.78], *P* < 0.001) and was evident in both females <55 years (OR = 0.59 [0.44–0.81], *P* < 0.001) and ≥55 years (OR = 0.74 [0.63–0.87], *P* < 0.001) with ACS. This difference was also evident in patients with stable angina where a reduced odds of higher potency agents were noted (OR = 0.78 [0.62–0.99], *P* = 0.044) (*Figure 3D*) and this was also evident in both age groups (*Figure 4*).

### Time trend analysis

**Figure 5:**
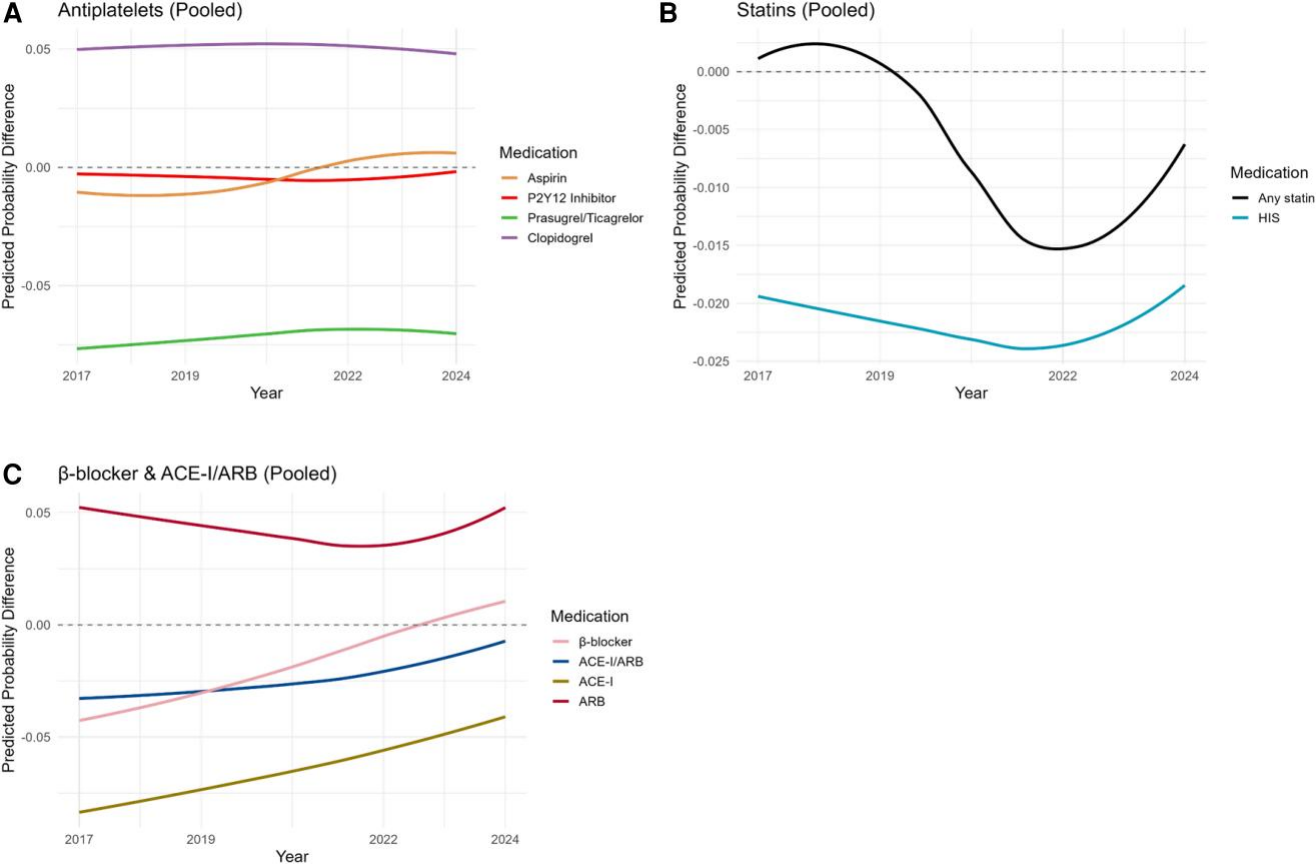
Time-based changes in prescribing of discharge medications between males and females in the pooled cohort, 2017–2024. (*A*) Antiplatelets (Aspirin *P* = 0.004, any P2Y12 inhibitor *P* = 0.985, Prasugrel/ticagrelor *P* = 0.645, Clopidogrel *P* = 0.942). (*B*) Statins (any statin *P* = 0.132, HIS *P* = 0.793). (*C*) β-blocker and ACE/ARB (β-blocker *P* = 0.033, ACE-I/ARB *P* = 0.402, ACE-I *P* = 0.219, ARB *P* = 0.573). Over time there was a narrowing in the gap in prescribing of β-blockers (*P* = 0.033) and aspirin (*P* = 0.003). No significant changes for other medications. Plotted values represent the difference in predicted probabilities over time between females and males. A negative difference suggests lower prescribing in females. Predicted probabilities were derived from joinpoint logistic regression modelling, allowing for as single breakpoint in time stratified by sex. Model was adjusted for age, BMI, hypertension, hypercholesterolaemia, previous MI, previous PCI, previous CABG, and current PCI.﻿.

**Figure 6:**
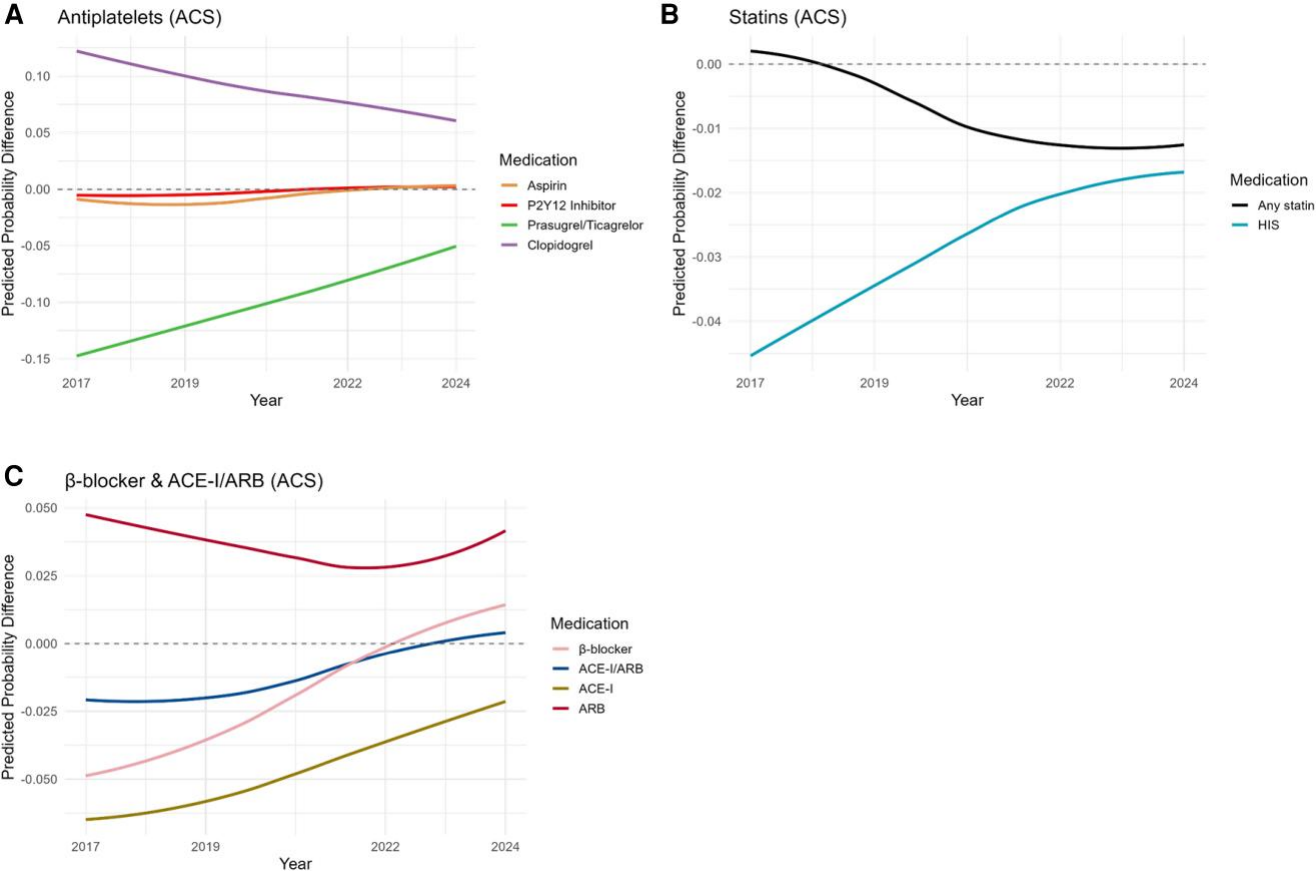
Time-based changes in prescribing of discharge medications between males and females in ACS, 2017–2024. (*A*) Antiplatelets (Aspirin *P* = 0.169, any P2Y12 inhibitor *P* = 0.395, Prasugrel/ticagrelor *P* = 0.085, Clopidogrel *P* = 0.220). (*B*) Statins (any statin *P* = 0.228, HIS *P* = 0.186). (*C*) β-blocker and ACE/ARB (β-blocker *P* = 0.018, ACE-I/ARB *P* = 0.269, ACE-I *P* = 0.242, ARB *P* = 0.481). Over time there was a narrowing in the gap in prescribing of β-blockers (*P* = 0.018) and a trend towards a narrowing in the gap of prescribing higher potency antiplatelet agents (*P* = 0.085). No significant changes for other medications. Plotted values represent the difference in predicted probabilities over time between females and males. A negative difference suggests lower prescribing in females. Predicted probabilities were derived from joinpoint logistic regression modelling, allowing for as single breakpoint in time stratified by sex. Model was adjusted for age, BMI, hypertension, hypercholesterolaemia, previous MI, previous PCI, previous CABG, and current PCI.

Joinpoint logistic regression analysis was carried out to assess changes in prescribing trends over time. Within the pooled all patient cohort (*Figure 5*), there was a significant increase in aspirin (*P* = 0.004) (*Figure 5A*) and β-blocker (*P* = 0.033) (*Figure 5C*) prescription over time in females compared to males with joinpoints post-COVID in 2021 and 2022, respectively. There were no significant changes in other medications over the study period. In the ACS sub-group (*Figure 6*), there is an apparent trend towards a reduction in the gap of prescribing higher potency P2Y_12_ inhibitors (*P* = 0.085) (*Figure 6A*) with an estimated joinpoint in 2022. Furthermore, whilst there was an apparent improvement in HIS prescriptions this was associated with a reverse trend in overall statin prescribing over time although this did not reach statistical significance (*Figure 6B*). A narrowing of the gap in β-blocker prescribing over time (*P* = 0.018) (*Figure 6C*) was clear with the estimated joinpoint in 2020. There were no significant changes in prescribing over time between the sexes in the stable angina group (see Supplementary material online, *Figure S2*).

### Sensitivity analysis

Additional relevant covariates were assessed in a sensitivity analysis. In the pooled group (see Supplementary material online, *Figure S1*) there was a statistically significant association between systolic blood pressure (<120 mmHg) and a lower odds of β-blocker prescribing in females compared to males (OR = 0.56 [0.37–0.85], *P* = 0.006) (see Supplementary material online, *Figure S3B*). A heart rate <60 b.p.m. was also associated with reduced prescribing of HIS (OR = 0.55 [0.31–0.98], *P* = 0.041) (see Supplementary material online, *Figure S3C*). In the ACS group, no statistically significant associations were observed between sex and LVEF and systolic blood pressure, haemoglobin, and eGFR in the context of our primary model for any medication class (Figure S4A, B and D). Interestingly, females with lower heart rate were significantly less likely to receive a P2Y_12_ inhibitor compared to males (OR = 0.23 [0.09–0.63], *P* = 0.004), however no other statistically significant associations were observed between sex and heart rate (see Supplementary material online, *Figure S4C*). In stable angina (see Supplementary material online, *Figure S5*), a heart rate <60 b.p.m. was also associated with reduced prescribing of HIS similar to seen in the pooled group (OR = 0.39 [0.17–0.90], *P* = 0.027) (see Supplementary material online, *Figure S5B*). Readmission status was not associated with any significant changes in the main results of the primary analysis (see Supplementary material online, *Table S9*).

### Ethnicity

To determine whether intersectionality might play a role in any of the differences observed, a further sub-analysis based on ethnicity was conducted for the whole cohort and within the ACS and stable angina groups. Overall, in the whole cohort (see Supplementary material online, *Figure S6*), fewer OMT drugs were prescribed to females in comparison to males in the Caucasian (see Supplementary material online, *Figure S6B*) and Asian groups (see Supplementary material online, *Figure S6C and D*). Focusing on the ACS group alone indicates that the reduced odds of HIS prescriptions in females is predominantly observed in the Caucasian population (see Supplementary material online, *Figure S7B*) (OR = 0.68 [0.50–0.92], *P* = 0.013). Additionally, the lower use of high potency P2Y_12_ inhibitors compared to males was seen in both the Caucasian and Asian groups (see Supplementary material online, *Figure S7B, C, and D*). There were no differences between the sexes in patients of black ethnicity (see Supplementary material online, *Figure S7A*) in ACS. Fewer differences were seen in the stable angina cohort (see Supplementary material online, *Figure S8*).

### Index of multiple deprivation

Analysis of sex differences according to IMD in both pooled (see Supplementary material online, *Figure S9*) and in ACS cohorts (see Supplementary material online, *Figure S10*) revealed that sex differences in higher potency P2Y_12_ inhibitors was most present in Quintiles 1–3 and not present in groups with lower deprivation scores, Quintiles 4–5. For HIS, we only saw sex differences in Quintile 4 (see Supplementary material online, *Figure S9D and S10D*). Fewer notable trends were observed across IMD groups in the stable angina cohort (see Supplementary material online, *Figure S11*).

## Discussion

There is a substantial evidence base indicating a region agnostic (e.g. USA,^22^ Australia,^23^ China,^24^ UK,^25^ Spain^26^) under-prescribing of OMT for women post-PCI that has been implicated in the increased risk for further events for women. This study provides novel insights into these disparities, suggesting a narrowing of the gap between the sexes in best practice prescribing post-2020. We assessed prescriptions post-PCI in consecutive attending patients in a busy and major tertiary cardiac centre serving a large ethnically diverse population (2.5 m people) in a part of London with some of the highest deprivation scores in the UK. In this study, we show that women continue to be prescribed several OMT drugs less frequently at discharge following angiography. Statistically significant differences in the prescribing of β-blockers, higher potency antiplatelet agents, and HIS in ACS and particularly NSTEMI patients played a prominent role in these differences. Time trend analysis, however, indicates an encouraging narrowing of the gap in OMT prescribing for women with statistically significant improvements in the prescription of β-blockers and trends towards improvements in high potency antiplatelet agents post-2020. These results indicate a promising improvement in the provision of OMT for women post-PCI but also highlight the need for continued vigilance and audit in this arena.

β-blocker prescribing at discharge was reduced in women compared to men in the pooled cohort overall and in those with ACS, as has been reported previously.^7,10,27^ However, disaggregation of the data by age, as an exploratory surrogate for confirmed menopausal status, and diagnosis suggested that these differences are driven predominantly in women <55 years and in those with NSTEMI. Data from 48 118 patients in the SWEDEHEART registry from 1994 to 2014 similarly showed reduced β-blocker prescribing in women <60 years, however differences were primarily observed in STEMI rather than NSTEMI patients.^28^ Why the two studies would be different is uncertain; however, the patients assessed in the Swedeheart study were from the Västra Götaland County, primarily populated by ethnic Swedes with smaller proportions of people of Finnish descent and from the middle east. In contrast, this London cohort is only ∼45% Caucasian with a large Asian heritage population and substantially higher levels of deprivation. These differences highlight potential issues related to bias.

Because of the above-described differences in β-blocker prescribing, we assessed whether cardiac function and/or blood pressure parameters might have led to the reduced prescribing in women. For this analysis we used LVEF, HR, and SBP as surrogates for testing these potential relationships, since sex differences in all three are well documented.^29^ In this dataset, whilst women had a lower incidence of abnormal LVEF, lower SBP and higher HR, sensitivity analysis showed that the sex differences in β-blocker prescriptions remained after adjustment for these in the whole cohort and in ACS. The role of β-blockers as a long-term therapy post ACS in those with normal LVEF has recently come under scrutiny, with the REDUCE-AMI study (∼22% female) suggesting that avoidance of β-blockers caused no significant detriment on composite cardiovascular outcomes, but the AβYSS study (∼17% female) suggesting continued benefit of therapy.^30,31^ In addition, whilst Reboot-CNC (19% female) showed no benefit in patients with MI and LVEF above 40%, BETAMI-DANBLOCK (20% female) showed reduced MACE in this group.^32,33^ Importantly, the number of women in these studies was typical of most ACS studies with low proportions relative to men and the data has not been segregated by sex. With respect to the latter, despite calls for trials to be stratified by sex and the data to be discussed with a sex-specific lens, the field has not been responsive with these issues remaining unaddressed in more recent trials.^34^ Women were more likely to have normal LVEF in the cohort compared to men, and the sensitivity analysis indicated that LVEF did not drive the sex differences. However, it should be noted that the analysis was exploratory with limited data points. It is possible that women may be receiving these medications without benefit but that cannot be confirmed by this analysis. Our data suggests that there has been a narrowing in the gap of prescribing of β-blockers over time, which appeared to correspond to pre- and post-COVID pandemic. This was a time when sex differences and health inequities came particularly under scrutiny with major calls for action.^1,35^ It is possible that the improvements evidenced in the current study have occurred consequent to this increasing awareness.

We found no difference in the overall prescriptions of aspirin and P2Y_12_ inhibitors between men and women. However, after angiography women were less likely to receive higher potency antiplatelet agents (prasugrel or ticagrelor) and more likely to receive lower potency antiplatelet agents (clopidogrel) compared to men. Similar differences have also been reported elsewhere in a large UK-based STEMI registry which included 168 181 patients.^36^ In our cohort, differences were primarily observed in patients with ACS, but again as with the β-blockers this was most prevalent in patients with NSTEMI, rather than STEMI, and evident in both pre- and post-menopausal women. Women in general have a higher bleeding risk following PCI compared to males, therefore avoidance of higher potency agents may be reflection of a greater perceived bleeding risk in women.^37^ The CRUSADE score was developed to stratify bleeding risk after NSTEMI and may be used in decision-making for antiplatelet regimens.^38^ CRUSADE scoring includes female sex as a risk factor for bleeding in itself. Furthermore, women have lower average baseline haematocrit, lower creatinine clearance, lower blood pressure, and higher heart rate all of which confer the potential for an increased CRUSADE score.^29,39,40^ Whilst the inclusion of females in the dataset on which this score is based was relatively good (40%), the resultant variables in the scoring system are not sex stratified which could potentially lead to an overestimation of bleeding risk in women, although this has not been examined in the literature.

Over time, there was a significant increase in the prescribing of β-blockers and a trend towards increased prescribing of higher potency antiplatelet agents. The reasons for this are not directly discernible from this analysis; however, increased awareness may be playing a key role in closing the gap, at least in this large cardiac centre in London, particularly since no major changes in guidelines around these medications occurred during this period. As female ACS patients tend to be older with more comorbidities, and this has been established to lead to increased bleeding risk after PCI, it may be challenging to close this gap within current treatment mandates. One important consideration is that much of the trial data available on OMT drugs relate to males in middle-age, and this has led to proposal of the argument that doses of antithrombotic drugs are not entirely appropriate for older women.^41^ However, a large meta-analysis showed that there were no significant sex differences in the efficacy and safety of high potency P2Y_12_ inhibitors compared to clopidogrel between the sexes. It should be noted that this analysis included only RCTs, where the participants are less likely to include older and co-morbid patients. Critically, women are more likely to be older and co-morbid when they have an event, reflected in this BartsRevasc cohort, so the results may not be truly representative of real-world populations of female patients.^42^ Better risk stratification for specific age groups may be particularly valuable in attempting to improve higher potency P2Y_12_ inhibitor use.

There was a pattern of reduced ACE-I and increased ARB prescribing in women that occurred across our cohort, albeit as a combined category most sex differences disappeared. Bradykinin induced cough from ACE-I is a common intolerance which occurs in 4–35% of patients,^43^ and women experience a higher incidence of ACE-I-related cough.^44^Guidelines advocate switching to an ARB if there is intolerance to ACE-I^11^ and it is likely that this mandate underlies in part the differences observed. Adverse drug reactions play an important role in medication-taking behaviour and long-term adherence, therefore avoiding predictable adverse drug reactions is an important consideration.^45^ This calls into question if there is a need for better identification of those women likely to suffer side effects from ACE-I and whether they should be initiated on ARB therapy from the start, as ARBs are generally thought to be non-inferior.^46^ Both ACE-I and ARBs are also teratogenic in pregnancy which may result in a reluctance to use either agent in women of childbearing age.^47^ Of note in women <55 in the pooled cohort a reduced combined ACE-I/ARB prescription was evident, but separation out by diagnosis demonstrated that this difference pertained to a reduction in women <55 years with stable angina. ACE-I/ARB prescriptions in stable angina are generally only recommended when other indications co-exist such as heart failure or hypertension or in patients with a very high risk of cardiovascular events.^48^ In this cohort, more women presented with hypertension but a lower incidence of heart failure. Thus, the reduced prescription in these women only shows a partial correlation with well-known co-morbidities and indicates likely some bias.

Whilst there were no differences between the sexes in the prescription of ‘any’ statin across groups, women ≥55 years with ACS were less likely to receive HIS.^11^ Wilkinson *et al*.^10^ found similarly reduced HIS prescriptions in women at discharge in secondary care in a large national UK cohort of MI patients from 2013 to 2017, however this wasn’t confined to a specific age group. A recent, large scale, Dutch study in primary care of 82 714 individuals showed that in new statin users, women were less likely to be prescribed HIS, both in patients with and without a history of cardiovascular disease, and women were less likely to achieve low-density lipoprotein treatment targets.^49^ Peters *et al*.^18^ studied prescriptions filled within 30 days after discharge for MI, finding women were less likely to fill HIS prescriptions compared to males, which was most notable in the youngest and oldest adults. In a population of adults with premature atherosclerotic cardiovascular disease, reduced HIS prescriptions in women were found 14–100 days from index primary care visit.^9^ Other non-sex specific analyses have found elderly populations are particularly less likely to receive appropriate statin therapy.^50^ Underlying reduced HIS prescriptions is the persistent perception that the elderly are at increased risk of muscle-related side effects, resulting in reduced use of statins in this group compared to younger counterparts; however, a large-scale meta-analysis did not support this view.^51^ It is noteworthy that whilst our time-based analysis suggests improvements in prescribing for women for other OMT drugs post-COVID, no significant improvements were achieved in HIS or ‘any’ statin therapy over time. A time-based analysis of primary care statin prescriptions by Kiss *et al*.^49^ showed that between 2011 and 2020 there had been an overall increase in statin prescribing in both sexes however the gap between males and females remained. Narrowing the statin gap should therefore be an urgent imperative for focus.

Exploratory socio-demographic sub-analysis according to IMD and ethnicity revealed that both these factors appear to influence prescribing. The sex differences in higher vs. lower potency P2Y_12_ inhibitors were observed across Quintiles 1–3 of deprivation but not in less deprived groups (Quintiles 4–5). A large-scale meta-analysis shows that whilst lower socio-economic status is associated with increased cardiovascular risk in both sexes, the association is stronger in women.^52,53^ Lower socio-economic status was also associated with increased initiation of clopidogrel over higher potency agents, in a large retrospective analysis of 55 664 patients undergoing PCI in the USA, although sex was not a significant determinate of differences.^54^ With respect to ethnicity, fewer women who were Caucasian and South Asian received the higher vs. the lower potency P2Y_12_ inhibitors in this London cohort, although this difference was not evident for black women. Importantly, black women made up a relatively small proportion of the cohort, so drawing firm conclusions is challenging. This differs from HIS where sex differences were primarily concentrated in the Caucasian population. Furthermore, unlike P2Y_12_ inhibitors differences primarily occurred in a higher socio-economic group (Quintile 4), although this was a small group and therefore again, findings should be interpreted with caution. It is possible that age may have been a factor since at least for the Caucasians, these individuals also had an older average age. With regards to ACE-I/ARBs, South Asian women were less likely to receive these medications compared to their male counterparts. As mentioned, women are at higher risk of adverse drug reactions from ACE-I, but in addition, Asians (not limited to South Asian) are also at increased risk of ACE-I induced cough, and this double perception of risk may underlie these differences, although a switch to ARB use would have been expected but was not evident in this dataset.^55^ Further studies on prescribing in South Asian populations is needed to understand what might underlie these differences. Unpicking the potential socio-demographic influencers of OMT sex differences is challenging due to the presence of multiple confounders and the challenge of generalizing findings to other populations. The results shown herein suggest there may be socio-demographic influencers even in a publicly funded health system such as the NHS, and we suggest that this should be taken into consideration when designing sex-specific guidance.

No significant differences in OMT prescribing in STEMI were observed in this cohort, likely as STEMI cohorts are homogeneous with more strictly protocolized care pathways and with less variability in practice compared to NSTEMI. Numerous observational studies have reported improvements in disparities following the introduction of care protocols. Improvements span the care pathway in CAD and include door to balloon times, medical therapies, and cardiovascular outcomes.^56,57^ In stable angina, differences reflected that of differential ACE-I vs. ARB use which was described above, otherwise there were no major differences noted. Much of the management in stable angina is guided by clinical situation such as severity of symptoms, need to stent, degree of stenosis, and overall cardiovascular risk profile—interpreting differences in this cohort is therefore more challenging and subject to more confounders.^48^

### Limitations

This study is based on observational data of discharge prescriptions and therefore reflects clinician practice within a single large tertiary cardiac unit serving a highly ethnically diverse population in London. Consequently, these findings may not reflect longer-term management in primary care, medication adherence, or wider practice across other centres. In addition, this is a real-world cohort, with women comprising approximately 20% of the participants limiting statistical power and the reliability of conclusions drawn from smaller sub-groups. Highlighting one of the key challenges in sex-specific analyses of CAD populations. Although we explored several additional clinically relevant factors including heart rate, blood pressure, LVEF, haemoglobin, and eGFR, numerous other potential factors and co-morbidities involved could not be assessed and may have influenced prescribing decisions. Furthermore, the extensive sub-group analyses undertaken increase the possibility of false-positive findings. Finally, outcome data including MACE were not available for this cohort; therefore, while observed prescribing disparities are clinically important, their direct relationship to adverse outcomes in women with CAD cannot be established.

## Conclusions

Time analysis of prescriptions at discharge in patients who underwent invasive angiography between 2017 and 2024 indicates that sex differences have been narrowing with greater equity in the prescription particularly of β-blockers and higher potency antiplatelet agents. Despite this at discharge women with ACS were overall less likely than men to receive several OMT drugs which differed across different age groups. Exploratory analysis suggested that women <55 years and those with NSTEMI were less likely to receive β-blockers. Whereas women >55 years appeared to drive differences noted in reduced HIS prescriptions. A reduced likelihood of receiving higher potency antiplatelet agents also occurred in women with ACS, but particularly NSTEMI, and at all ages. Our observations add further granularity regarding sex differences in discharge medications in CAD sub-groups and highlight the need for initiatives to improve practice in the treatment particularly of NSTEMI patients. Whether the improvements observed in the practice of equitable prescribing in this large ethnically diverse cohort reflect improvements that might equally be observed across the globe is uncertain. We suggest that a review internationally, particularly comparing pre- and post-COVID, would be of value. This review should be coupled with an assessment of outcomes to ascertain whether improved practices where they exist lead to improved outcomes. The findings from such assessments are likely to identify relatively simple changes in practice that may prove beneficial for women.

Since prescribing guidelines did not change dramatically over the time period assessed, the observations intimate an enhanced awareness of inequitable treatment of women that drives correction in this large cardiac centre. Our findings suggest that regular formal assessment of male:female access to optimal medical therapy is likely to lead to improved quality in practices.

The findings highlight improvements in equitable prescription of medicines post-discharge following angiography. But also highlight the benefits and the need for continued vigilance and audit of prescribing practices to ensure that best practice guideline dictated care is equitably delivered.

## Supplementary Material

oeag099_Supplementary_Data

## Data Availability

Data analysed in this article will be made available on reasonable request to the corresponding author.

## Pre-registered clinical trial number

NCT05255705, EDGE ID: 142567.
