# Supplementary material for: Narrowing sex differences in pharmacotherapy following invasive angiography for coronary artery disease between 2017 and 2024
Source: Eur Heart J Open. 2026 Jun 8;6(4):oeag099. doi: 10.1093/ehjopen/oeag099 (PMC13353530; doi:10.1093/ehjopen/oeag099)
Supplement: oeag099_Supplementary_Data [file oeag099_supplementary_data.pdf]

Supplement

Supplementary Figures and Tables

Narrowing sex differences in pharmacotherapy following invasive angiography for coronary artery disease between 2017 and 2024

Andrew J Sullivan[1, 2], Zin Tun[2], Margaret Siu[1], Manisha Rai[1], Gloria Antoun[1], Nandini Rawat[1], Rebecca Walker[1], Alexandros Kythreotis[1], Abdelmajid Al Jariri[2], Asad Shabbir[1], Shane Cashin[2], Anthony Mathur[1, 2], Daniel A Jones[1, 2], Ajay Gupta[1], Krishnaraj Rathod[1, 2], Amrita Ahluwalia[1]

[1] Barts and the London Faculty of Medicine and Dentistry, Queen Mary University of London

[2] Barts Heart Centre, Barts Health NHS Trust, London

Author for Correspondence:

Prof Amrita Ahluwalia

Barts and the London Faculty of Medicine and Dentistry,

Charterhouse Square

Queen Mary University of London

London

EC1M 6BQ

Email: a.ahluwalia@qmul.ac.uk

Table S1. Demographics of the ACS cohort. Statistics are expressed as number and % in brackets of the group for categorical data. For continuous data mean ± SD is reported. Categorical data were compared using the Chi-squared test. Normally distributed continuous data were compared using a t test and non-normally distributed continuous data compared using the Mann-Whitney U test.

	**All**	**Female**	**Male**	**P value**
Patients	5844	1314 (22.5%)	4530 (77.5%)	
Age	61.7 (±12.7)	66.1 (±12.7)	60.4 (±12.4)	<0.001
**Ethnicity**				
Black	308 (5.3%)	96 (7.3%)	212 (4.7%)	<0.001
Caucasian	2640 (45.2%)	649 (49.4%)	1991 (44.0%)	0.001
South Asian	1573 (26.9%)	323 (24.6%)	1250 (27.6%)	0.033
Other Asian	558 (9.5%)	91 (6.9%)	467 (10.3%)	<0.001
Mixed	85 (1.5%)	23 (1.8%)	62 (1.4%)	0.375
Not stated	680 (11.6%)	132 (10.0%)	548 (12.1%)	0.046
**Co-morbidities**				
Hypertension	3226 (55.2%)	837 (63.7%)	2389 (52.7%)	<0.001
Hypercholesterolaemia	2947 (50.4%)	698 (53.1%)	2249 (49.6%)	0.085
Diabetes	2087 (36.2%)	561 (42.9%)	1526 (34.3%)	<0.001
Previous MI	1336 (22.9%)	259 (19.7%)	1077 (23.8%)	0.008
Previous PCI	1269 (21.7%)	242 (18.4%)	1027 (22.7%)	0.003
Previous CABG	345 (5.9%)	67 (5.1%)	278 (6.1%)	0.131
**Intervention**				
PCI with stent	4180 (71.5%)	917 (69.8%)	3263 (72.0%)	0.121
PCI with no stent	503 (8.6%)	122 (9.3%)	381 (8.4%)	0.348
**LV ejection fraction**				
≥50%	2286 (39.1%)	534 (40.6%)	1752 (38.7%)	0.211
30-49%	1131 (19.4%)	254 (19.3%)	877 (19.4%)	1.000
<30%	373 (6.4%)	73 (5.6%)	300 (6.6%)	0.184
Not stated	2054 (35.1%)	453 (34.5%)	1601 (35.3%)	0.584
**Clinical measurements**				
BMI	27.8 (±6.2)	28.0 (±6.0)	27.8 (±6.3)	0.373
Heart rate (bpm)	71.04 (±11.29)	71.56 (±11.10)	70.89 (±11.35)	0.191
Systolic blood pressure (mmHg)	119.5 (±18.1)	119.1 (±18.4)	119.6 (±18.5)	0.594
Diastolic blood pressure (mmHg)	69.5 (±10.0)	66.7 (±9.7)	70.2 (±10.0)	<0.001

Table S2. Demographics of the STEMI cohort. Statistics are expressed as number and % in brackets of the group for categorical data. For continuous data mean ± SD is reported. Categorical data were compared using the Chi-squared test. Normally distributed continuous data were compared using a t test and non-normally distributed continuous data compared using the Mann-Whitney U test.

	**All**	**Female**	**Male**	**P value**
Patients	2127	432 (20.3%)	1695 (79.7%)	
Age	59.6 (±12.4)	65.5 (±12.8)	58.1 (±11.9)	<0.001
**Ethnicity**				
Black	112 (5.3%)	35 (8.1%)	77 (4.5%)	0.005
Caucasian	1012 (47.6%)	232 (53.7%)	780 (46.%0)	0.005
South Asian	469 (22.0%)	69 (16.0%)	400 (23.6%)	0.001
Other Asian	220 (10.3%)	33 (7.6%)	187 (11.0%)	0.048
Mixed	35 (1.6%)	7 (1.6%)	28 (1.7%)	1.000
Not stated	279 (13.1%)	56 (13.0%)	223 (13.2%)	0.979
**Co-morbidities**				
Hypertension	900 (42.3%)	227 (52.5%)	673 (39.7%)	<0.001
Hypercholesterolaemia	746 (35.1%)	163 (37.7%)	583 (34.4%)	0.429
Diabetes	553 (26.7%)	136 (31.8%)	417 (25.4%)	0.010
Previous MI	258 (12.1%)	40 (9.3%)	218 (12.9%)	0.106
Previous PCI	268 (12.6%)	43 (10.0%)	225 (13.3%)	0.161
Previous CABG	37 (1.7%)	5 (1.2%)	32 (1.9%)	0.377
**Intervention**				
PCI with stent	1890 (88.9%)	379 (87.7%)	1511 (89.1%)	0.455
PCI with no stent	147 (6.9%)	33 (7.6%)	114 (6.7%)	0.574
**LV ejection fraction**				
≥50%	439 (20.6%)	80 (18.5%)	359 (21.2%)	0.249
30-49%	423 (19.9%)	84 (19.4%)	339 (20.0%)	0.849
<30%	123 (5.8%)	24 (5.6%)	99 (5.8%)	0.911
Not stated	1142 (53.7%)	244 (56.5%)	898 (53.0%)	0.212
**Clinical measurements**				
BMI	27.7 (±5.7)	27.8 (±5.4)	27.7 (±5.8)	0.690
Heart rate (bpm)	71.9 (±10.9)	72.42 (±10.4)	71.76 (±11.0)	0.439
Systolic blood pressure (mmHg)	115.6 (±16.2)	115.86 (±17.0)	115.5 (±16.0)	0.784
Diastolic blood pressure (mmHg)	68.6 (±10.2)	66.1 (±9.4)	69.3 (±10.3)	<0.001

Table S3. Demographics of the NSTEMI cohort. Statistics are expressed as number and % in brackets of the group for categorical data. For continuous data mean ± SD is reported. Categorical data were compared using the Chi-squared test. Normally distributed continuous data were compared using a t test and non-normally distributed continuous data compared using the Mann-Whitney U test.

	All	Female	Male	P value
Patients	3717	882 (23.7%)	2835 (76.3%)	
Age	62.9 (±12.7)	66.3 (±12.7)	61.8 (±12.5)	<0.001
**Ethnicity**				
Black	196 (5.3%)	61 (6.9%)	135 (4.8%)	0.016
Caucasian	1628 (43.8%)	417 (47.3%)	1211 (42.7%)	0.019
South Asian	1104 (29.7%)	254 (28.8%)	850 (30.0%)	0.529
Other Asian	338 (9.1%)	58 (6.6%)	280 (9.9%)	0.004
Mixed	50 (1.3%)	16 (1.8%)	34 (1.2%)	0.224
Not stated	401 (10.8%)	76 (8.6%)	325 (11.5%)	0.020
**Co-morbidities**				
Hypertension	2326 (62.6%)	610 (69.2%)	1716 (60.5%)	<0.001
Hypercholesterolaemia	2201 (59.2%)	535 (60.7%)	1666 (58.8%)	0.596
Diabetes	1534 (41.5%)	425 (48.2%)	1109 (39.4%)	<0.001
Previous MI	1078 (29.0%)	219 (24.8%)	859 (30.3%)	0.006
Previous PCI	1001 (26.9%)	199 (22.6%)	802 (28.3%)	0.002
Previous CABG	308 (8.3%)	62 (7.0%)	246 (8.7%)	0.174
**Intervention**				
PCI with stent	2290 (61.6%)	538 (61.0%)	1752 (61.8%)	0.698
PCI with no stent	356 (9.6%)	89 (10.1%)	267 (9.4%)	0.598
**LV ejection fraction**				
≥50%	1847 (49.7%)	454 (51.5%)	1393 (49.1%)	0.240
30-49%	708 (19.0%)	170 (19.3%)	538 (19.0%)	0.883
<30%	250 (6.7%)	49 (5.6%)	201 (7.1%)	0.131
Not stated	912 (24.5%)	209 (23.7%)	703 (24.8%)	0.536
**Clinical measurements**				
BMI	27.9 (±6.5)	28.0 (±6.3)	27.8 (±6.6)	0.445
Heart rate (bpm)	70.6 (±11.5)	71.1 (±11.4)	70.4 (±11.5)	0.232
Systolic blood pressure (mmHg)	121.6 (±18.8)	120.7 (±18.8)	121.9 (±18.8)	0.234
Diastolic blood pressure (mmHg)	69.9 (±9.9)	67.0 (±9.9)	70.8 (±9.7)	<0.001

Table S4. Demographics of the stable angina cohort. Statistics are expressed as number and % in brackets of the group for categorical data. For continuous data mean ± SD is reported. Categorical data were compared using the Chi-squared test. Normally distributed continuous data were compared using a t test and non-normally distributed continuous data compared using the Mann-Whitney U test.

	**All**	**Female**	**Male**	**P value**
Patients	4747	1046 (22.0%)	3701 (78.0%)	
Age	63.4 (±11.1)	66.0 (±10.6)	62.7 (±11.1)	<0.001
**Ethnicity**				
Black	223 (4.7%)	84 (8.0%)	139 (3.8%)	<0.001
Caucasian	2147 (45.2%)	491 (46.9%)	1656 (44.7%)	0.221
South Asian	1390 (29.3%)	270 (25.8%)	1120 (30.3%)	0.006
Other Asian	354 (7.5%)	65 (6.2%)	289 (7.8%)	0.096
Mixed	41 (0.9%)	11 (1.1%)	30 (0.8%)	0.579
Not stated	592 (12.5%)	125 (12.0%)	467 (12.6%)	0.600
**Co-morbidities**				
Hypertension	3024 (63.7%)	698 (66.7%)	2326 (62.8%)	0.023
Hypercholesterolaemia	3194 (67.3%)	683 (65.3%)	2511 (67.8%)	0.233
Diabetes	1847 (39.0%)	472 (45.2%)	1375 (37.3%)	<0.001
Previous MI	1735 (36.5%)	280 (26.8%)	1455 (39.3%)	<0.001
Previous PCI	1978 (41.7%)	330 (31.5%)	1648 (44.5%)	<0.001
Previous CABG	549 (11.6%)	78 (7.5%)	471 (12.7%)	<0.001
**Intervention**				
PCI with stent	2862 (60.3%)	630 (60.2%)	2232 (60.3%)	0.992
PCI with no stent	382 (8.0%)	67 (6.4%)	315 (8.5%)	0.032
**LV ejection fraction**				
≥50%	2738 (57.7%)	627 (59.9%)	2111 (57.0%)	0.100
30-49%	583 (12.3%)	81 (7.7%)	502 (13.6%)	<0.001
<30%	121 (2.5%)	15 (1.4%)	106 (2.9%)	0.013
Not stated	1305 (27.5%)	323 (30.9%)	982 (26.5%)	0.006
**Clinical measurements**				
BMI	28.1 (±5.8)	28.4 (±6.5)	28.1 (±5.5)	0.158
Heart rate (bpm)	67.8 (±11.1)	69.3 (±10.6)	67.4 (±11.1)	0.011
Systolic blood pressure (mmHg)	126.8 (±19.6)	128.6 (±21.3)	126.4 (±19.1)	0.088
Diastolic blood pressure (mmHg)	71.2 (±9.7)	69.4 (±9.2)	71.7 (±9.7)	<0.001

Table S5. Unadjusted medication prescription at discharge following invasive angiography by sex in ACS. Statistics are expressed as the absolute number and % in brackets of the group. Chi-squared test was used to determine differences between groups.

Medication	All	Female	Male	P value
**Aspirin**	5534 (94.7)	1228 (93.5)	4306 (95.1)	0.027
**P2Y12 inhibitor**	5465 (93.5)	1216 (92.5)	4249 (93.8)	0.118
Clopidogrel	2603 (44.5)	716 (54.5)	1887 (41.7)	<0.001
Prasugrel & Ticagrelor	3044 (52.1)	541 (41.2)	2503 (55.3)	<0.001
**Any statin**	5610 (96.0)	1249 (95.1)	4361 (96.3)	0.053
High intensity statin	5244 (89.7)	1137 (86.5)	4107 (90.7)	<0.001
**β-blocker**	5242 (89.7)	1146 (87.2)	4096 (90.4)	0.001
**ACE-I or ARB**	5058 (86.6)	1112 (84.6)	3946 (87.1)	0.022
ACE-I	4341 (74.3)	901 (68.6)	3440 (76.0)	<0.001
ARB	780 (13.3)	225 (17.1)	555 (12.3)	<0.001

Table S6. Unadjusted medication prescription at discharge following invasive angiography by sex in STEMI. Statistics are expressed as the absolute number and % in brackets of the group. Chi-squared test was used to determine differences between groups.

Medication	All	Female	Male	P value
**Aspirin**	2015 (94.7)	402 (93.1)	1613 (95.2)	0.103
**P2Y12 inhibitor**	2017 (94.8)	403 (93.3)	1614 (95.2)	0.134
Clopidogrel	467 (22.0)	131 (30.3)	336 (19.8)	<0.001
Prasugrel & Ticagrelor	1589 (74.7)	280 (64.8)	1309 (77.2)	<0.001
**Any statin**	2034 (95.6)	409 (94.7)	1625 (95.9)	0.341
High intensity statin	1911 (89.8)	369 (85.4)	1542 (91.0)	0.001
**β-blocker**	1953 (91.8)	379 (87.7)	1574 (92.9)	0.001
**ACE-I or ARB**	1955 (91.9)	388 (89.8)	1567 (92.4)	0.090
ACE-I	1768 (83.1)	345 (79.9)	1423 (84.0)	0.051
ARB	209 (9.8)	50 (11.6)	159 (9.4)	0.202

Table S7. Unadjusted medication prescription at discharge following invasive angiography by sex in NSTEMI. Statistics are expressed as the absolute number and % in brackets of the group. Chi-squared test was used to determine differences between groups.

Medication	All	Female	Male	P value
**Aspirin**	3519 (94.7)	826 (93.7)	2693 (95.0)	0.144
**P2Y12 inhibitor**	3448 (92.8)	813 (92.2)	2635 (92.9)	0.487
Clopidogrel	2136 (57.5)	585 (66.3)	1551 (54.7)	<0.001
Prasugrel & Ticagrelor	1455 (39.1)	261 (29.6)	1194 (42.1)	<0.001
**Any statin**	3576 (96.2)	840 (95.2)	2736 (96.5)	0.094
High intensity statin	3333 (89.7)	768 (87.1)	2565 (90.5)	0.005
**β-blocker**	3289 (88.5)	767 (87.0)	2522 (89.0)	0.112
**ACE-I or ARB**	3103 (83.5)	724 (82.1)	2379 (83.9)	0.212
ACE-I	2573 (69.2)	556 (63.0)	2017 (71.2)	<0.001
ARB	571 (15.4)	175 (19.8)	396 (14.0)	<0.001

Table S8. Unadjusted medication prescription at discharge following invasive angiography by sex in stable angina. Statistics are expressed as the absolute number and % in brackets of the group. Chi-squared test was used to determine differences between groups.

Medication	All	Female	Male	P value
**Aspirin**	4233 (89.2)	921 (88.0)	3312 (89.5)	0.205
**P2Y12 inhibitor**	3515 (74.0)	741 (70.8)	2774 (75.0)	0.008
Clopidogrel	2820 (59.4)	640 (61.2)	2180 (58.9)	0.196
Prasugrel & Ticagrelor	777 (16.4)	110 (10.5)	667 (18.0)	<0.001
**Any statin**	4352 (91.7)	952 (91.0)	3400 (91.9)	0.398
High intensity statin	3919 (82.6)	838 (80.1)	3081 (83.2)	0.021
**β-blocker**	3606 (76.0)	761 (72.8)	2845 (76.9)	0.007
**ACE-I or ARB**	3102 (65.3)	639 (61.1)	2463 (66.5)	0.001
ACE-I	2262 (47.7)	411 (39.3)	1851 (50.0)	<0.001
ARB	859 (18.1)	239 (22.8)	620 (16.8)	<0.001

Table S9. Sensitivity analysis for readmission status. Odds ratios, 95% confidence intervals and P values are displayed from the primary analysis and sensitivity analysis using only index admissions to assess whether in-patient clustering had any significant bearing on displayed results. Where the odds value or P value change in the index admission analysis this is highlighted.

Group	Medication	Primary analysis	Index admission analysis	OR direction change	P value change	N= primary analysis	N= index admission analysis
**Pooled**	Aspirin	0.88 (0.729-1.067), p=0.186	0.915 (0.745-1.133), p=0.408	No	No	10591	9446
	Any $P2Y_{12}$	0.878 (0.755-1.024), p=0.096	0.897 (0.762-1.058), p=0.196	No	No	10591	9446
	Clopidogrel	1.244 (1.128-1.372), p=<0.001	1.259 (1.135-1.396), p=<0.001	No	No	10591	9446
	Prasugrel/ ticagrelor	0.72 (0.644-0.805), p=<0.001	0.721 (0.64-0.812), p=<0.001	No	No	10591	9446
	Any statin	0.846 (0.693-1.041), p=0.107	0.896 (0.715-1.134), p=0.350	No	No	10589	9444
	High intensity statin	0.877 (0.759-1.017), p=0.079	0.907 (0.776-1.066), p=0.230	No	No	10591	9446
	β-blocker	0.819 (0.722-0.931), p=0.002	0.825 (0.721-0.946), p=0.005	No	No	10590	9446
	ACE or ARB	0.837 (0.749-0.937), p=0.002	0.843 (0.749-0.95), p=0.005	No	No	10590	9445
	ACE	0.745 (0.675-0.823), p=<0.001	0.752 (0.677-0.835), p=<0.001	No	No	10590	9445
	ARB	1.356 (1.188-1.546), p=<0.001	1.346 (1.168-1.548), p=<0.001	No	No	10591	9446
**ACS**	Aspirin	0.865 (0.647-1.176), p=0.339	0.919 (0.675-1.276), p=0.600	No	No	5844	5553
	Any P2Y12	0.906 (0.697-1.19), p=0.467	0.919 (0.701-1.221), p=0.551	No	No	5844	5553
	Clopidogrel	1.407 (1.227-1.614), p=<0.001	1.378 (1.196-1.588), p=<0.001	No	No	5844	5553
	Prasugrel/ ticagrelor	0.683 (0.592-0.789), p=<0.001	0.712 (0.614-0.827), p=<0.001	No	No	5844	5553
	Any statin	0.828 (0.669-1.034), p=0.090	0.827 (0.663-1.041), p=0.098	No	No	5843	5552
	High intensity statin	0.778 (0.574-1.072), p=0.115	0.776 (0.565-1.087), p=0.129	No	No	5844	5553
	β-blocker	0.78 (0.636-0.962), p=0.018	0.788 (0.637-0.98), p=0.030	No	No	5843	5552
	ACE or ARB	0.854 (0.71-1.033), p=0.099	0.892 (0.734-1.089), p=0.254	No	No	5843	5553
	ACE	0.779 (0.672-0.905), p=<0.001	0.789 (0.677-0.923), p=0.003	No	No	5843	5552
	ARB	1.267 (1.045-1.528), p=0.015	1.301 (1.063-1.582), p=0.009	No	No	5844	5553
**STEMI**	Aspirin	0.995 (0.596-1.778), p=0.985	0.962 (0.576-1.722), p=0.890	No	No	2127	2091
	Any P2Y12	0.881 (0.546-1.492), p=0.618	0.857 (0.53-1.454), p=0.547	No	No	2127	2091
	Clopidogrel	1.175 (0.875-1.56), p=0.274	1.118 (0.825-1.497), p=0.461	No	No	2127	2091
	Prasugrel/ ticagrelor	0.828 (0.63-1.101), p=0.186	0.857 (0.647-1.146), p=0.289	No	No	2127	2091
	Any statin	0.865 (0.522-1.514), p=0.591	0.84 (0.506-1.471), p=0.518	No	No	2127	2091
	High intensity statin	0.787 (0.55-1.155), p=0.204	0.769 (0.536-1.129), p=0.164	No	No	2127	2091

	β-blocker	0.77 (0.52-1.175), p=0.207	0.775 (0.522-1.189), p=0.223	No	No	2127	2091
	ACE or ARB	0.846 (0.578-1.274), p=0.406	0.923 (0.62-1.421), p=0.705	No	No	2127	2091
	ACE	0.933 (0.696-1.267), p=0.651	0.95 (0.703-1.301), p=0.742	No	No	2127	2091
	ARB	1.048 (0.709-1.513), p=0.807	1.105 (0.744-1.604), p=0.608	No	No	2127	2091
**NSTEMI**	Aspirin	0.79 (0.555-1.146), p=0.200	0.868 (0.592-1.309), p=0.481	No	No	3717	3462
	Any P2Y12	0.907 (0.665-1.256), p=0.547	0.936 (0.676-1.318), p=0.696	No	No	3717	3462
	Clopidogrel	1.439 (1.218-1.702), p=<0.001	1.43 (1.202-1.704), p=<0.001	No	No	3717	3462
	Prasugrel/ ticagrelor	0.651 (0.544-0.779), p=<0.001	0.677 (0.561-0.817), p=<0.001	No	No	3717	3462
	Any statin	0.732 (0.5-1.094), p=0.117	0.731 (0.487-1.127), p=0.142	No	No	3716	3461
	High intensity statin	0.852 (0.654-1.123), p=0.244	0.856 (0.648-1.147), p=0.286	No	No	3717	3462
	β-blocker	0.816 (0.642-1.043), p=0.099	0.822 (0.64-1.066), p=0.132	No	No	3716	3462
	ACE or ARB	0.879 (0.712-1.091), p=0.235	0.904 (0.724-1.136), p=0.379	No	No	3716	3462
	ACE	0.747 (0.629-0.889), p=<0.001	0.752 (0.628-0.904), p=0.002	No	No	3717	3462
	ARB	1.344 (1.074-1.671), p=0.009	1.37 (1.08-1.725), p=0.008	No	No	3716	3462
**Stable angina**	Aspirin	0.892 (0.696-1.154), p=0.375	0.9 (0.68-1.204), p=0.469	No	No	4747	3893
	Any P2Y12	0.842 (0.677-1.047), p=0.122	0.858 (0.668-1.101), p=0.229	No	No	4747	3893
	Clopidogrel	1.027 (0.864-1.223), p=0.760	1.002 (0.822-1.222), p=0.986	No	No	4747	3893
	Prasugrel/ ticagrelor	0.782 (0.613-0.989), p=0.043	0.79 (0.587-1.05), p=0.111	No	**Yes**	4747	3893
	Any statin	0.906 (0.694-1.2), p=0.479	1.044 (0.757-1.472), p=0.800	**Yes**	No	4746	3892
	High intensity statin	0.939 (0.769-1.153), p=0.541	1.028 (0.822-1.296), p=0.813	**Yes**	No	4747	3893
	β-blocker	0.869 (0.736-1.03), p=0.102	0.903 (0.754-1.084), p=0.269	No	No	4747	3893
	ACE or ARB	0.862 (0.739-1.006), p=0.059	0.879 (0.745-1.039), p=0.129	No	No	4747	3893
	ACE	0.725 (0.624-0.842), p=<0.001	0.755 (0.641-0.887), p=<0.001	No	No	4747	3893
	ARB	1.422 (1.18-1.708), p=<0.001	1.361 (1.11-1.662), p=0.003	No	No	4747	3893

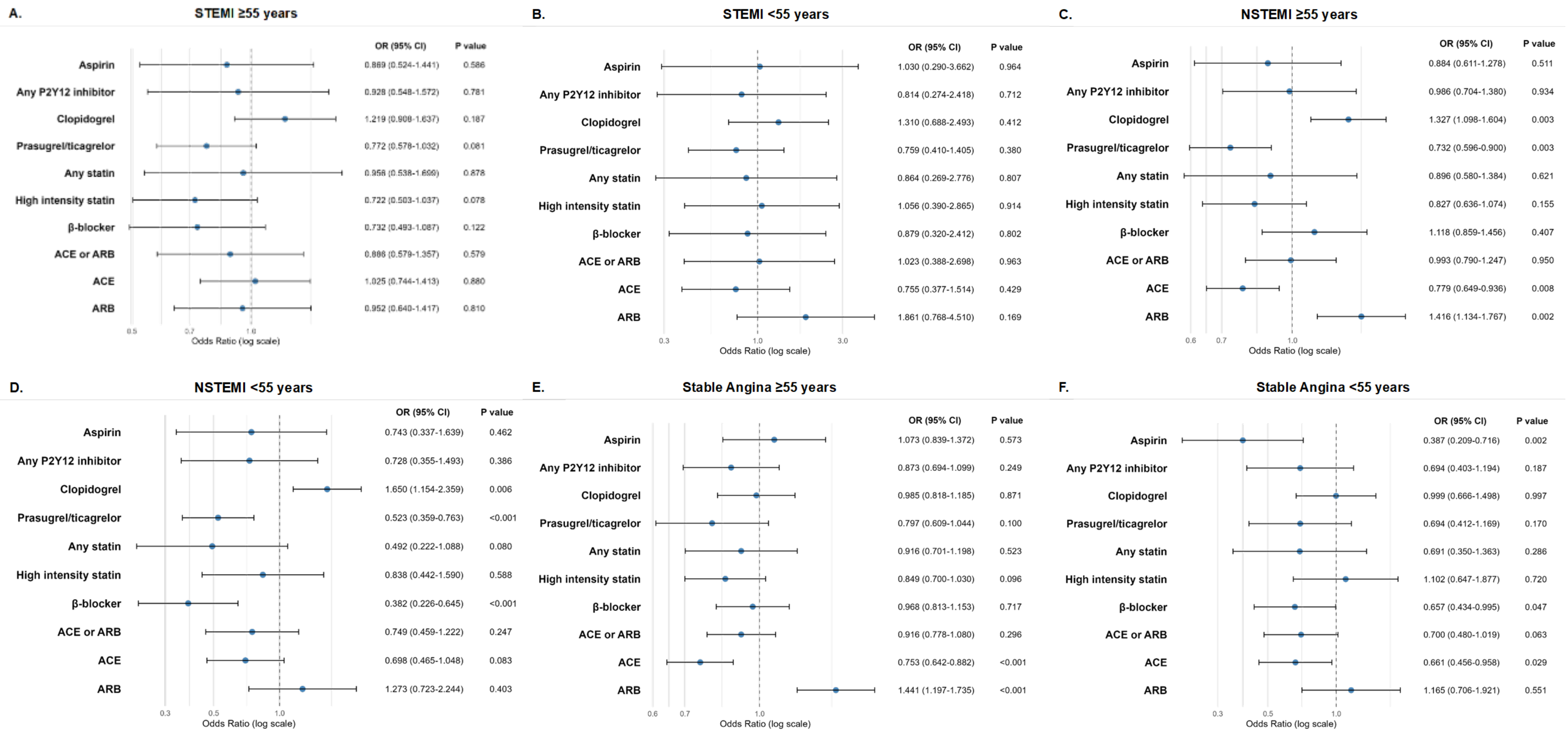

Figure S1. Multivariate logistic regression analysis* for sex differences in OMT according to age in stable angina. (A) STEMI patients ≥55 years (n= 1371, female=340). (B) STEMI patients <55 years (n= 756, female=92). **(C)** NSTEMI patients ≥55 years (n=2713, female=719). **(D)** NSTEMI patients <55 years (n=1004, female=163). **(E)** Stable angina patients ≥55 years (n=3697, female=887). **(F)** Stable angina patients <55 years (n=1050, female=159). Statistics are displayed as odds ratios and 95% confidence intervals. Reference population = males, therefore odds ratio <1 taken as in females. *adjusted for age, BMI, diabetes, hypertension, hypercholesterolaemia, previous MI, previous PCI, previous CABG and current PCI

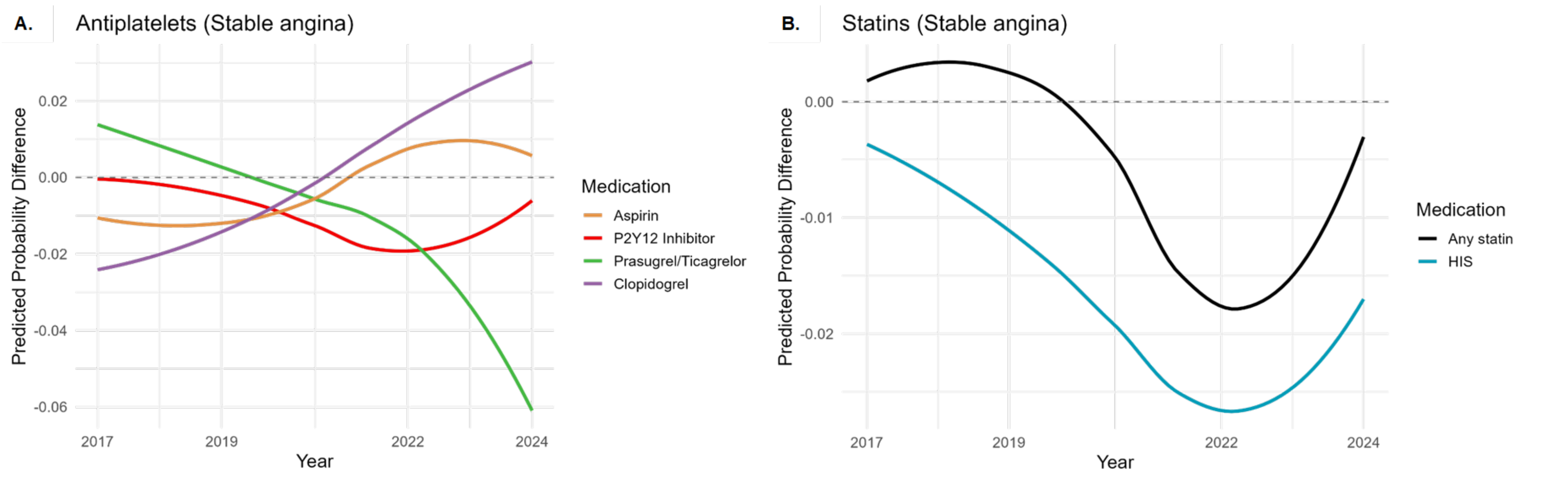

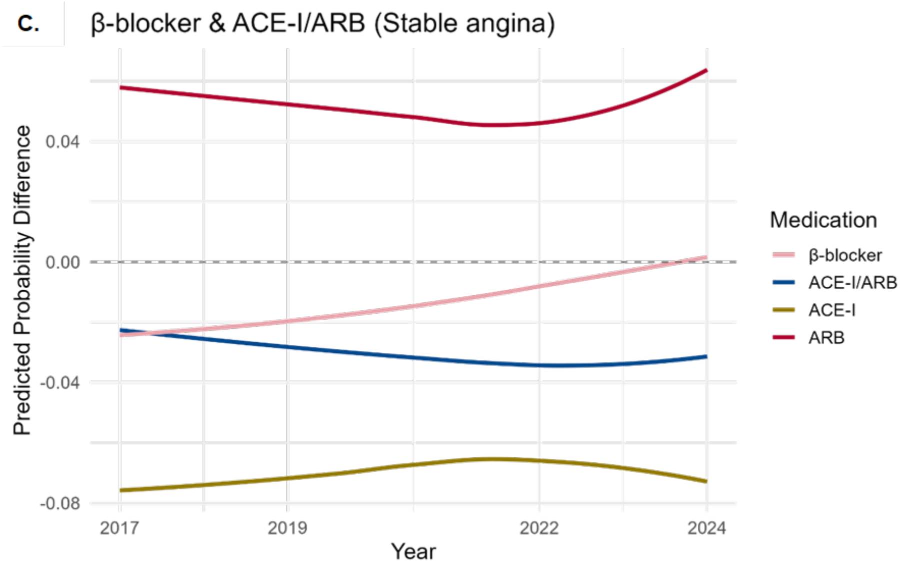

Figure S2. Time based changes in prescribing of discharge medications between males and females in stable angina, 2017-2024. **(A)** Antiplatelets (Aspirin p=0.008, Any P2Y12 inhibitor p=0.462, Prasugrel/ticagrelor p=0.021, Clopidogrel p=0.071). **(B)** Statins (Any statin p=0.279, HIS p=0.555). **(C)** β-blocker & ACE/ARB (β-blocker p=0.571, ACE-I/ARB p=0.844, ACE-I p=0.934, ARB p=0.946). There was a narrowing in the gap over time in prescribing of aspirin (p=0.008), however an increase in the gap of prescribing higher potency antiplatelet agents (p=0.021). Plotted values represent the difference in predicted probabilities over time between females and males. A negative difference suggests lower prescribing in females. Predicted probabilities were derived from joinpoint logistic regression modelling, allowing for as single breakpoint in time stratified by sex. Model was adjusted for age, BMI, hypertension, hypercholesterolaemia, previous MI, previous PCI, previous CABG and current PCI.

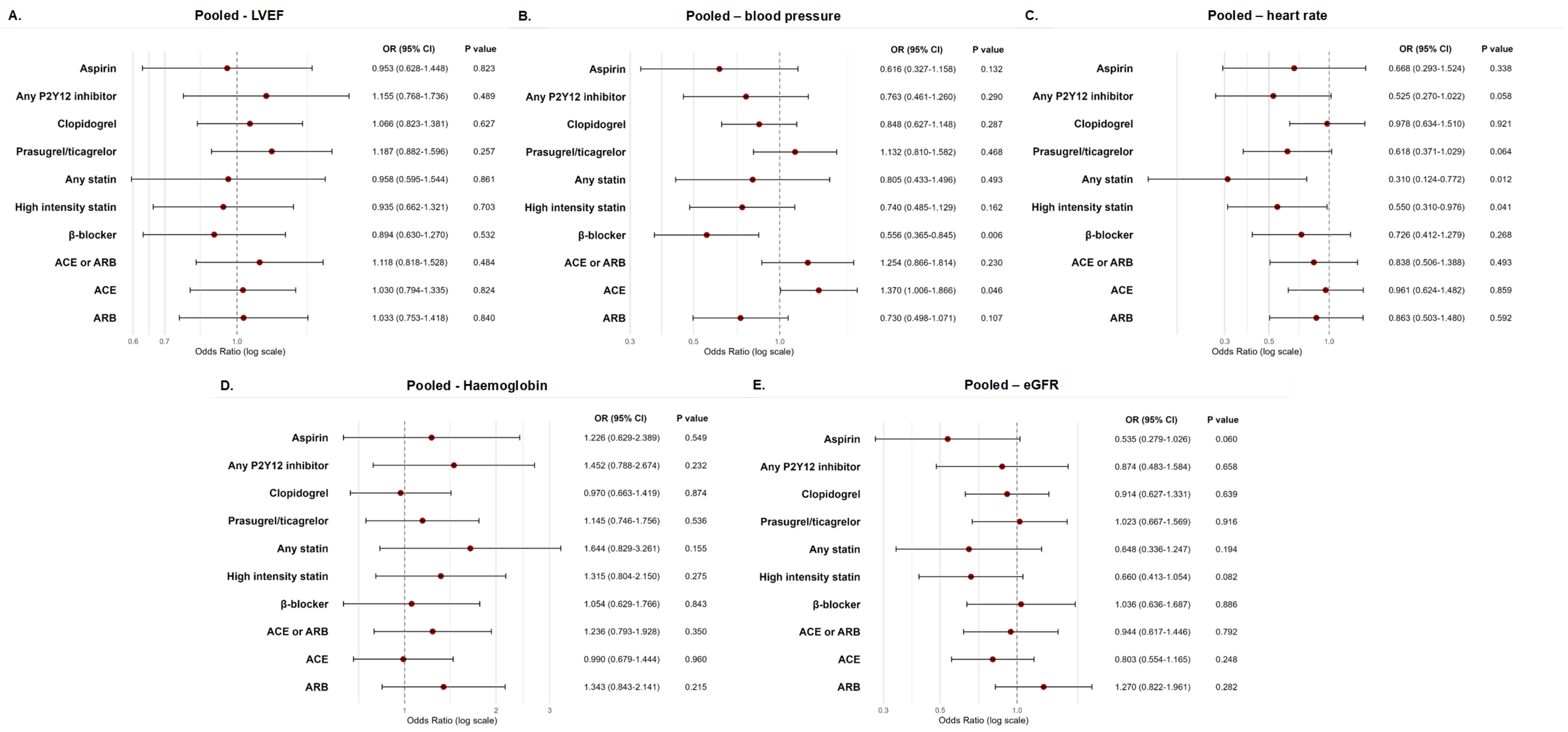

Figure S3. Sensitivity analysis of additional covariates in the pooled cohort. **(A)** LVEF (<50%). **(B)** Blood pressure (<120 mmHg). **(C)** Heart rate (<60 bpm). **(D)** Haemoglobin (<120 g/L). **(E)** eGFR (<60 mL/min/1.73m²). Statistics displayed represent odds ratio and 95% confidence interval of interaction factor between sex and relevant covariate. Reference population = males, therefore odds ratio <1 taken as reduced in females. Multivariate logisticregression was used for sensitivity analysis with the additional covariate included as an interaction factor in the main model.

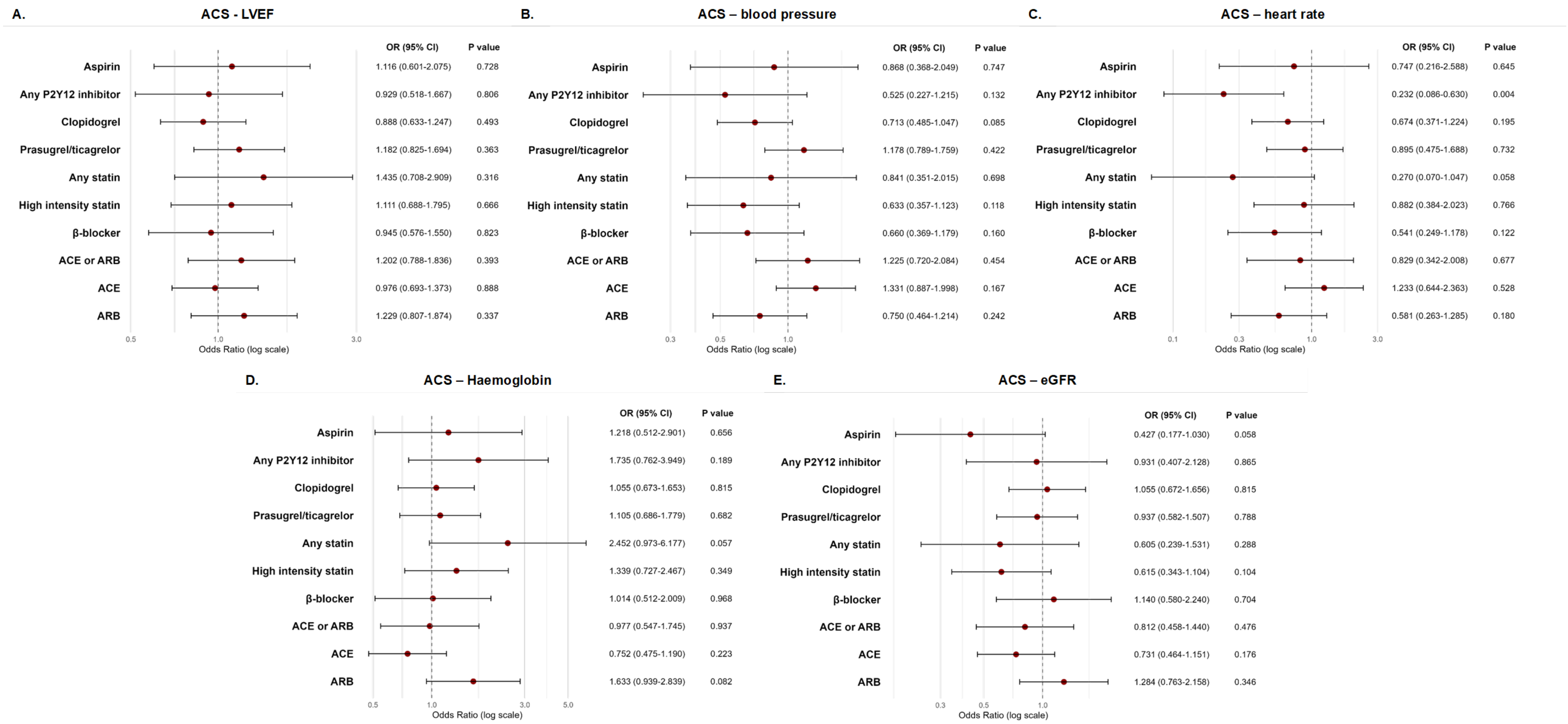

Figure S4. Sensitivity analysis of additional covariates in ACS. **(A)** LVEF (<50%). **(B)** Blood pressure (<120 mmHg). **(C)** Heart rate (<60 bpm). **(D)** Haemoglobin (<120 g/L). **(E)** eGFR (<60 mL/min/1.73m²). Statistics displayed represent odds ratio and 95% confidence interval of interaction factor between sex and relevant covariate. Reference population = males, therefore odds ratio <1 taken as reduced in females. Multivariate logistic regression was used for sensitivity analysis with the additional covariate included as an interaction factor in the main model.

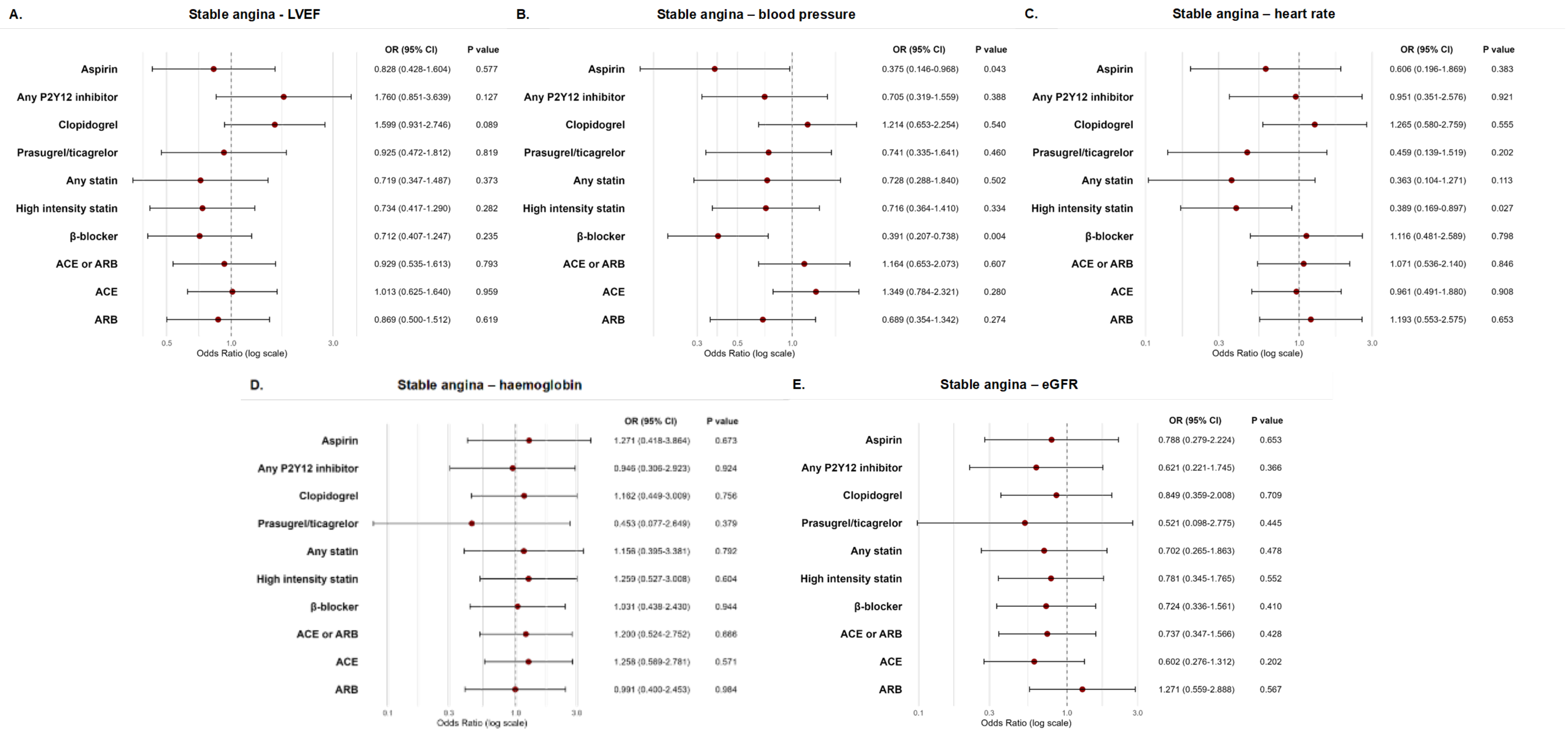

Figure S5. Sensitivity analysis of additional covariates in stable angina. **(A)** LVEF (<50%). **(B)** Blood pressure (<120 mmHg). **(C)** Heart rate (<60 bpm). **(D)** Haemoglobin (<120 g/L). **(E)** eGFR (<60 mL/min/1.73m²). Statistics displayed represent odds ratio and 95% confidence interval of interaction factor between sex and relevant covariate. Reference population = males, therefore odds ratio <1 taken as reduced in females. Multivariate logistic regression was used for sensitivity analysis with the additional covariate included as an interaction factor in the main model.

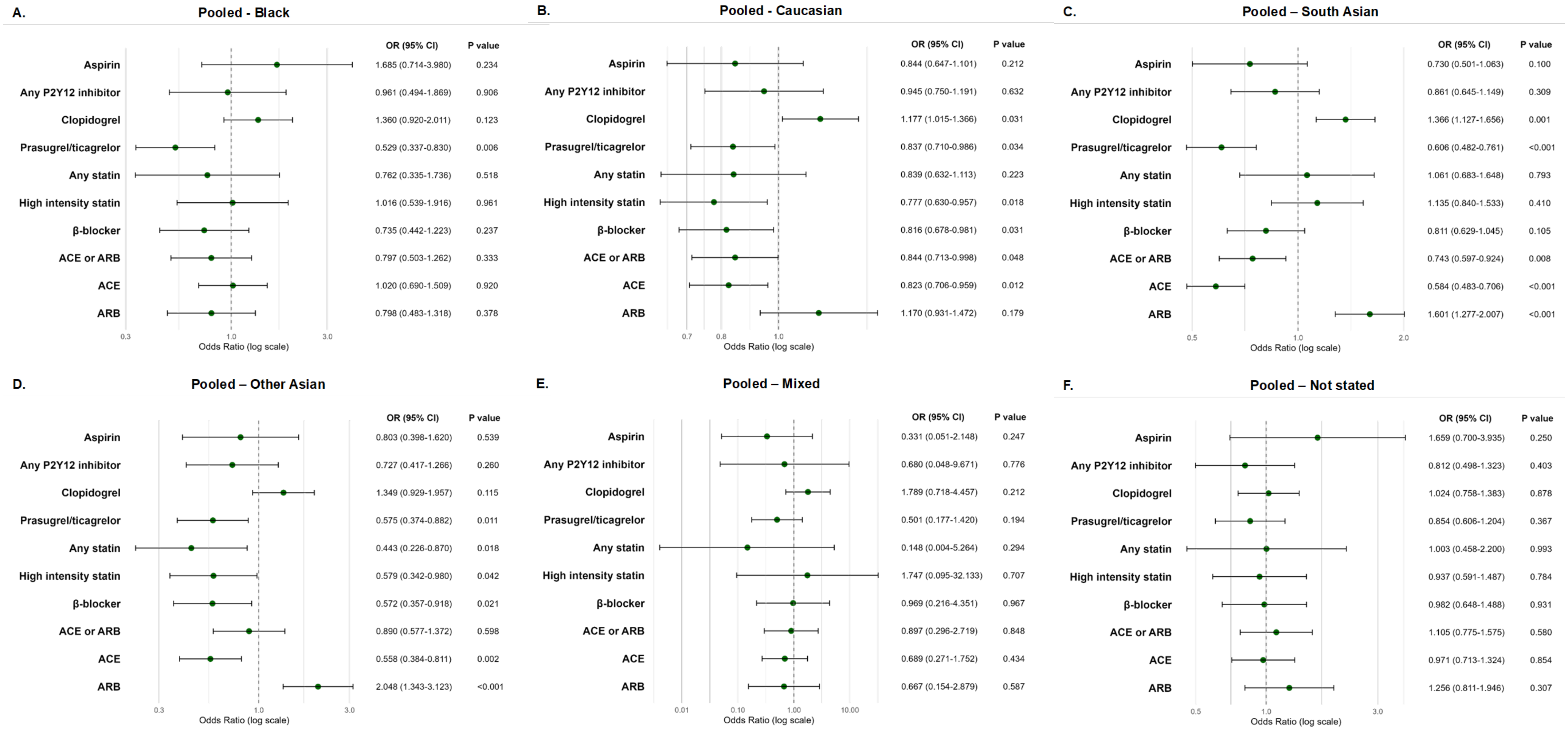

Figure S6. Multivariate logistic regression analysis* of sex differences in OMT according to ethnicity in the pooled cohort. **(A)** Black (n=531, female=180). **(B)** Caucasian (n=4787, female=1140). **(C)** South Asian (n=2963, female=593). **(D)** Other Asian (n=912, female=156). **(E)** Mixed ethnicity (n=126, female=34). **(F)** Ethnicity not stated (n=1272, female=257). *adjusted for age, BMI, diabetes, hypertension, hypercholesterolaemia, previous MI, previous PCI, previous CABG and current PCI

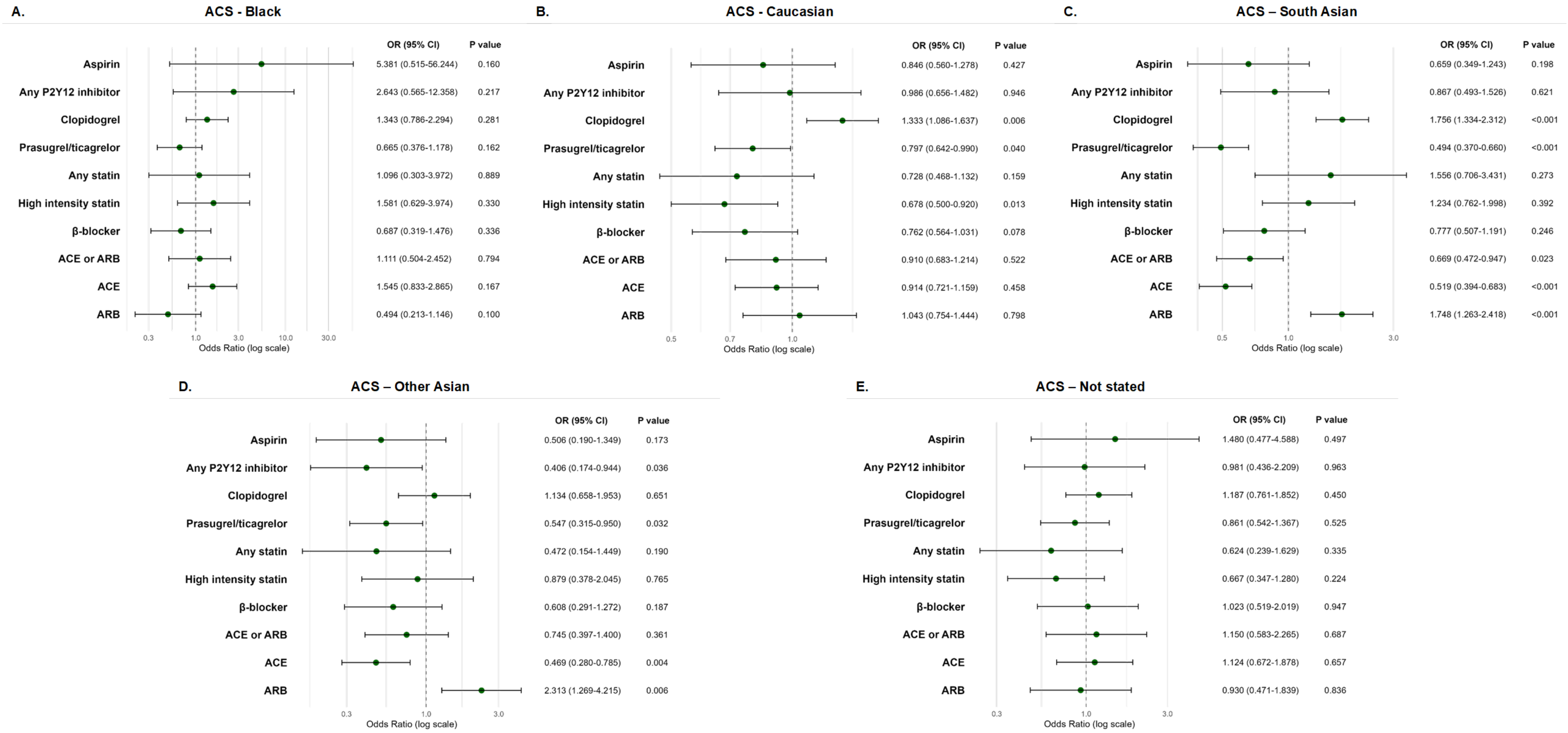

Figure S7. Multivariate logistic regression analysis* of sex differences in OMT according to ethnicity in the pooled cohort. **(A)** Black (n=308, female=96). **(B)** Caucasian (n=2640, female=649). **(C)** South Asian (n=1573, female=323). **(D)** Other Asian (n=558, female=91). **(E)** Ethnicity not stated (n=680, female=132). Note the 'Mixed ethnicity' group is not displayed due to insufficient sample size leading to unstable estimates and excessively wide confidence intervals. *adjusted for age, BMI, diabetes, hypertension, hypercholesterolaemia, previous MI, previous PCI, previous CABG and current PCI

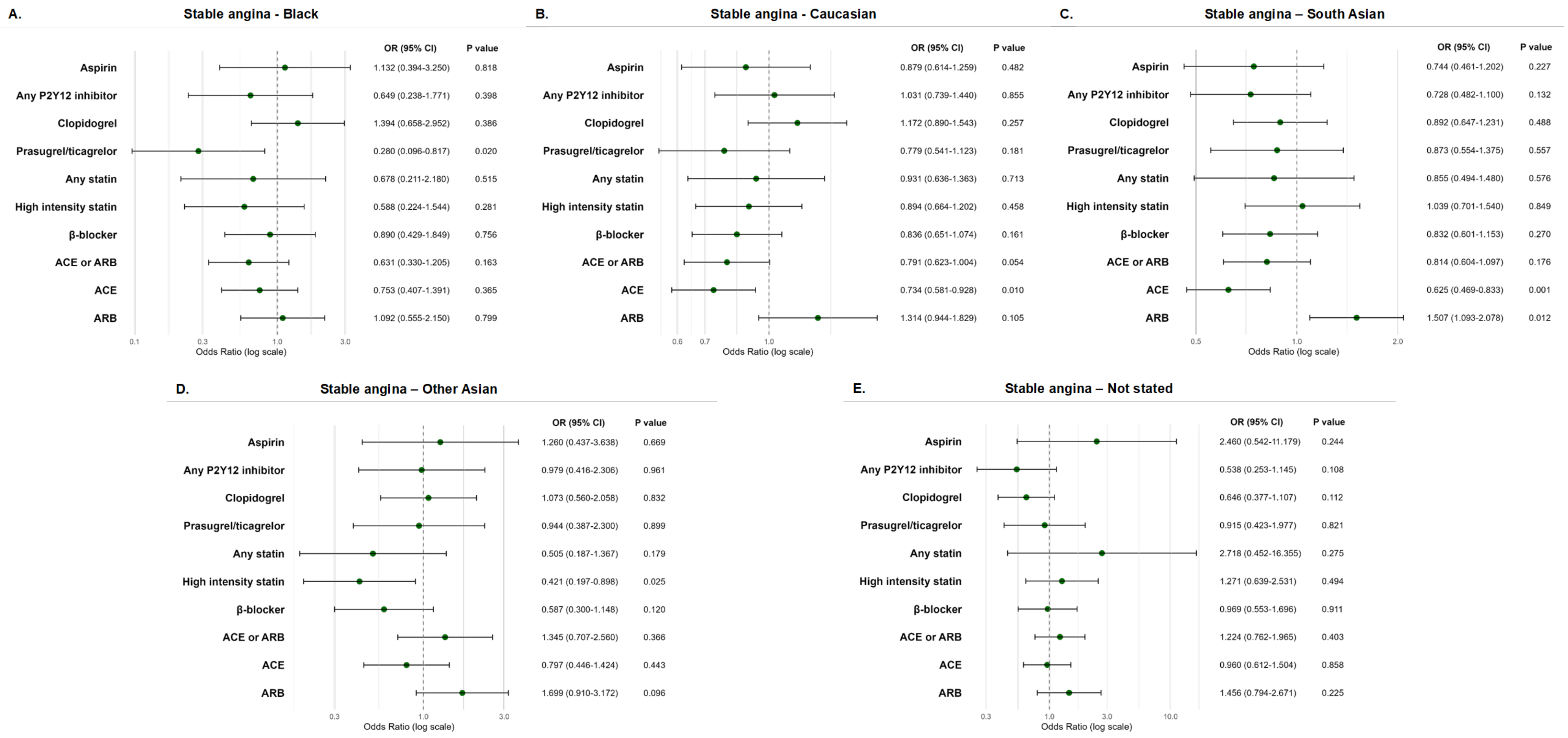

Figure S8. Multivariate logistic regression analysis* of sex differences in OMT according to ethnicity in the pooled cohort. **(A)** Black (n=223, female=84). **(B)** Caucasian (n=2147, female=491). **(C)** South Asian (n=1573, female=323). **(D)** Other Asian (n=354, female=65). **(E)** Ethnicity not stated (n=592, female=125). Note the 'Mixed ethnicity' group is not displayed due to insufficient sample size leading to unstable estimates and excessively wide confidence intervals. *adjusted for age, BMI, diabetes, hypertension, hypercholesterolaemia, previous MI, previous PCI, previous CABG and current PCI

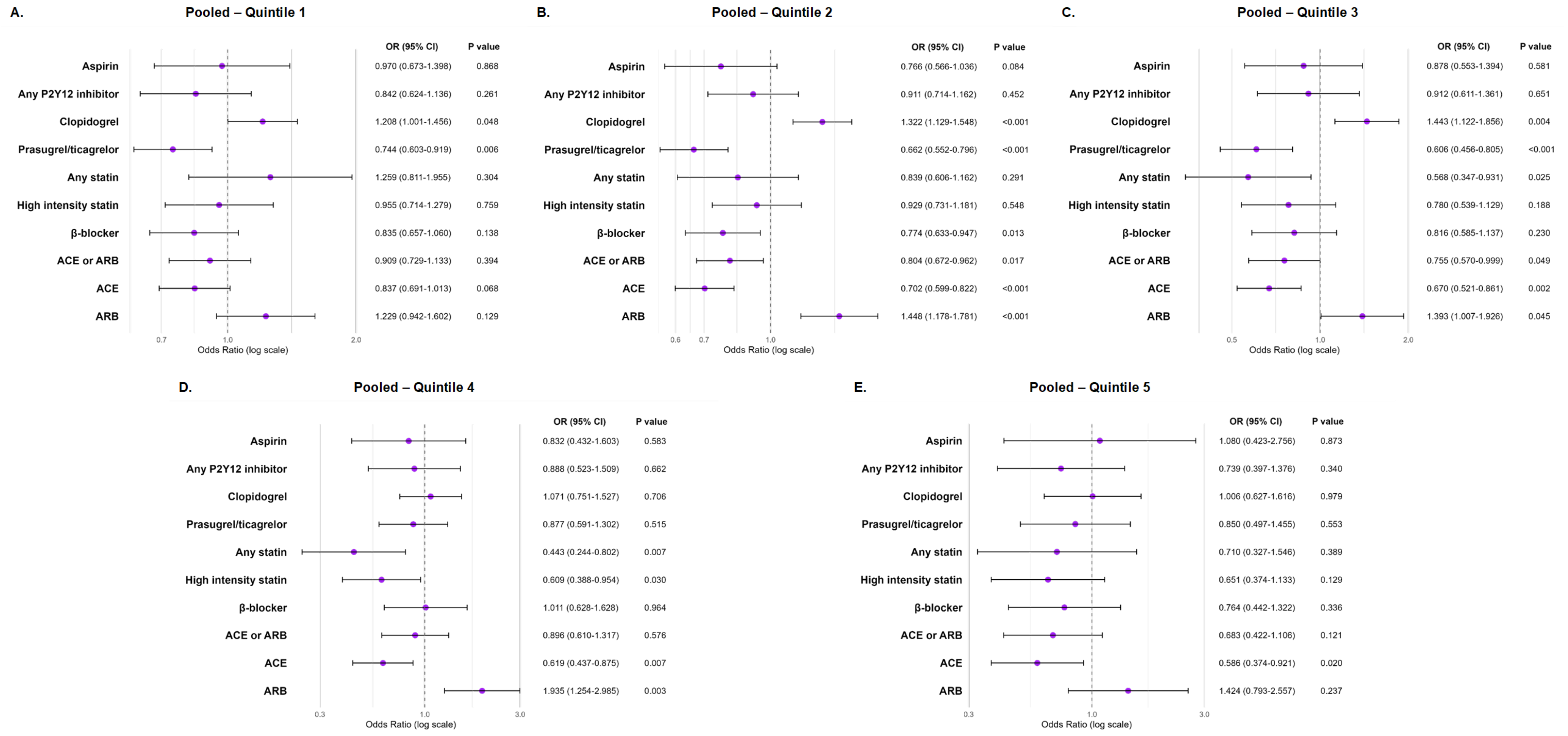

Figure S9. Multivariate logistic regression analysis* of sex differences in OMT according to IMD quintile in the pooled cohort. **(A)** Quintile 1 (n=2590, female=643). **(B)** Quintile 2 (n=4059, female=906). **(C)** Quintile 3 (n=1893, female=389). **(D)** Quintile 4 (n=1099, female=224). **(E)** Quintile 5 (n=759, female=163). IMD not available n=191. Quintile 1 represents highest deprivation score. *adjusted for age, BMI, diabetes, hypertension, hypercholesterolaemia, previous MI, previous PCI, previous CABG and current PCI

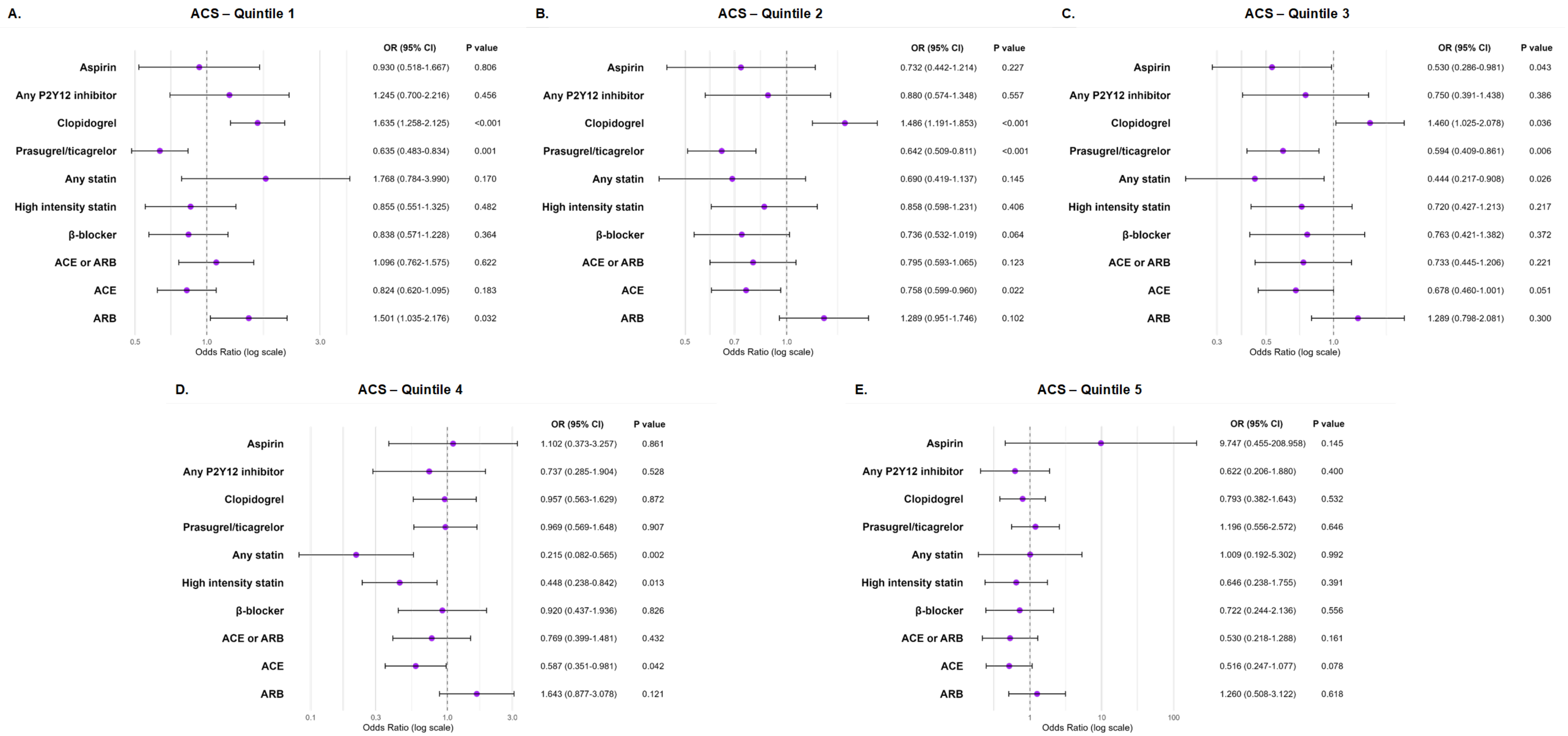

Figure S10. Multivariate logistic regression analysis* of sex differences in OMT according to IMD quintile in ACS. **(A)** Quintile 1 (n=1454, female=380). **(B)** Quintile 2 (n=2237, female=1074). **(C)** Quintile 3 (n=1009, female=803). **(D)** Quintile 4 (n=578, female=119). **(E)** Quintile 5 (n=384, female=78). IMD not available n=182. Quintile 1 represents highest deprivation score. *adjusted for age, BMI, diabetes, hypertension, hypercholesterolaemia, previous MI, previous PCI, previous CABG and current PCI

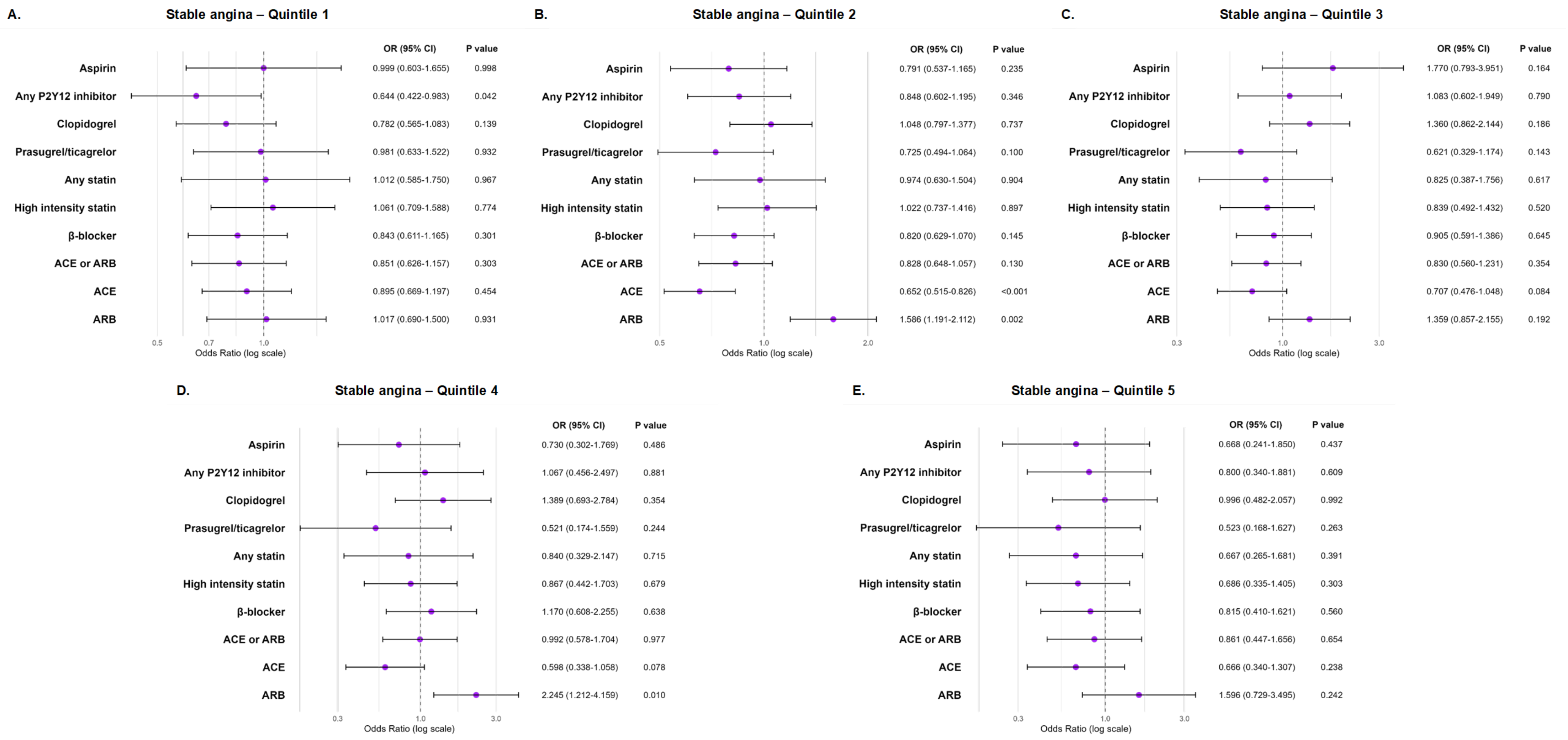

Figure S11. Multivariate logistic regression analysis* of sex differences in OMT according to IMD quintile in ACS. **(A)** Quintile 1 (n=1136, female=263). **(B)** Quintile 2 (n=1822, female=410). **(C)** Quintile 3 (n=884, female=183). **(D)** Quintile 4 (n=521, female=105). **(E)** Quintile 5 (n=375, female=85). IMD not available n=9. Quintile 1 represents highest deprivation score. *adjusted for age, BMI, diabetes, hypertension, hypercholesterolaemia, previous MI, previous PCI, previous CABG and current PCI
